# Possible Effect of the use of Mesenchymal Stromal Cells in the Treatment of Autism Spectrum Disorders: A Review

**DOI:** 10.3389/fcell.2022.809686

**Published:** 2022-07-05

**Authors:** Ryad Tamouza, Fernanda Volt, Jean-Romain Richard, Ching-Lien Wu, Jihène Bouassida, Wahid Boukouaci, Pauline Lansiaux, Barbara Cappelli, Graziana Maria Scigliuolo, Hanadi Rafii, Chantal Kenzey, Esma Mezouad, Soumia Naamoune, Leila Chami, Florian Lejuste, Dominique Farge, Eliane Gluckman

**Affiliations:** ^1^ Translational Neuropsychiatry, INSERM, IMRB, DMU, AP-HP, Univ Paris Est Créteil, Créteil, France; ^2^ Institut de Recherche Saint Louis (IRSL), Eurocord, Hôpital Saint Louis, Assistance Publique-Hôpitaux de Paris (AP-HP), Université Paris Cité, Paris, France; ^3^ Translational Neuropsychiatry, INSERM, IMRB, Univ Paris Est Créteil, Créteil, France; ^4^ Unité de Médecine Interne (UF 04), CRMR MATHEC, Maladies Auto-immunes et Thérapie Cellulaire, Centre de Référence des Maladies Auto-immunes Systémiques Rares D’Ile-de-France MATHEC, AP-HP, Hôpital St-Louis, Paris, France; ^5^ Monacord, Centre Scientifique de Monaco, Monaco, Monaco

**Keywords:** autism, mesenchymal stromal cells, cellular therapy, inflammation, immuno-modulation

## Abstract

Autism spectrum disorder (ASD) represents a set of heterogeneous neurodevelopmental conditions defined by impaired social interactions and repetitive behaviors. The number of reported cases has increased over the past decades, and ASD is now a major public health burden. So far, only treatments to alleviate symptoms are available, with still unmet need for an effective disease treatment to reduce ASD core symptoms. Genetic predisposition alone can only explain a small fraction of the ASD cases. It has been reported that environmental factors interacting with specific inter-individual genetic background may induce immune dysfunctions and contribute to the incidence of ASD. Such dysfunctions can be observed at the central level, with increased microglial cells and activation in ASD brains or in the peripheral blood, as reflected by high circulating levels of pro-inflammatory cytokines, abnormal activation of T-cell subsets, presence of auto-antibodies and of dysregulated microbiota profiles. Altogether, the dysfunction of immune processes may result from immunogenetically-determined inefficient immune responses against a given challenge followed by chronic inflammation and autoimmunity. In this context, immunomodulatory therapies might offer a valid therapeutic option. Mesenchymal stromal cells (MSC) immunoregulatory and immunosuppressive properties constitute a strong rationale for their use to improve ASD clinical symptoms. *In vitro* studies and pre-clinical models have shown that MSC can induce synapse formation and enhance synaptic function with consequent improvement of ASD-like symptoms in mice. In addition, two preliminary human trials based on the infusion of cord blood-derived MSC showed the safety and tolerability of the procedure in children with ASD and reported promising clinical improvement of core symptoms. We review herein the immune dysfunctions associated with ASD provided, the rationale for using MSC to treat patients with ASD and summarize the current available studies addressing this subject.

## 1 Introduction

Autism spectrum disorder (ASD) is a set of heterogeneous neurodevelopmental conditions with onset of symptoms within the first 3 years of life. It is characterized by repetitive behaviors, restricted range of activities, and impairments in social communication (King et al., 2014). As per the DSM-5 (Diagnostic and Statistical Manual of Mental Disorders) definition, ASD combines the formerly separated disorders of autism, Asperger’s syndrome, pervasive developmental disorders not otherwise specified, and childhood disintegrative disorder (American Psychiatric Association, 2013).

The number of ASD reported cases has increased over the past decades. The World Health Organization estimates that 1 in 270 people has ASD, with a prevalence 4 times higher in men compared to women (World Health Organization, 2021). The ASD incidence in Europe is, approximately, 1% with a total of 3 million patients at the present time. In the United States, a 30% increase of the pediatric prevalence of ASD was observed from 2012 to 2014 alone (Corcoran et al., 2015). It is, currently, estimated that ASD affects over 2 million individuals with approximately 1 in 68 American children identified as having ASD (World Health Organization, 2021).

In the most severe form, ASD is considered a chronic, disabling disorder that compromises the full potential of the affected individual. In the United States, the lifetime cost of supporting an individual with ASD is estimated between $1.4 and $2.4 million, depending on the degree and severity of involvement (Buescher et al., 2014). Beyond the financial aspect, ASD remains a condition that severely debilitates social integration and creates a major emotional burden for the patients’ families.

There is no currently accessible effective medical treatment to address ASD core symptoms such as communication impairment, social inadaptability or lack of empathy (Thompson Foundation for Autism, 2012). Therapeutic options include non-specific medications, as well as behavioral, occupational and speech therapies, and specialized educational and vocational support. Currently available treatments, such as psychotropic medications, are prescribed to improve irritability, seizures, mood disorders or hyperactive syndrome, but they are not disease-modifying compounds *per se* (Lai et al., 2014).

Although the exact etiology of ASD is unknown, recent studies have indicated that genetic and environmental factors contribute to the disease development (Chaste and Leboyer, 2012; Bennabi et al., 2015; Meltzer and Van de Water, 2017; Bennabi et al., 2018; Brown et al., 2018). In recent years, through genetic analysis and sequencing, *de novo* mutations, several genes copy number variants (CNV) and single-nucleotide polymorphisms (SNP) have been identified and were shown to increase the risk for ASD. Many of the affected genes, such as those encoding neuroligin-3, neurexin-1,2,3 (Vaags et al., 2012) multiple ankyrin repeat domains 3 (SHANK3) (Guilmatre et al., 2014) and contactin-associated protein-2 (CNTNAP2) (Scott-Van Zeeland et al., 2010) are known to regulate the synaptic function. Moreover, several ASD associated CNVs are enriched in genes required for normal synaptic transmission (Abrahams and Geschwind, 2010). A specific genetic cause for ASD can be identified in less than 20% of cases; in the remaining individuals, ASD development is likely attributed to complex interactions between multiple genetic and environmental factors. Among the environmental factors associated with an increased risk of ASD are prematurity, birth complications, maternal teratogen exposure, environmental toxins, and advanced parental age (Rylaarsdam and Guemez-Gamboa, 2019). In addition, inflammation and immune dysfunctions have been amply demonstrated to be implicated in ASD (Chaste and Leboyer, 2012; Bennabi et al., 2015; Meltzer and Van de Water, 2017; Bennabi et al., 2018; Brown et al., 2018).

In this context, immunoregulatory treatment approaches for ASD patients are appealing. Several studies have emphasized and demonstrated that the immunoregulatory and immunosuppressive properties of the mesenchymal stromal cells (MSCs) constitute an experimental rationale for the use of these cells to treat immune-mediated diseases. While their immunoregulatory properties have been primarily focused on their ability to inhibit the proliferation of T-lymphocytes, studies have shown that MSCs affect the function and the differentiation of several other types of immunocompetent cells (Di Nicola et al., 2002; Jiang et al., 2005; Corcione et al., 2006; Sotiropoulou et al., 2006).

This review aims to describe ASD, present an overview of the immune disfunctions that may be associated with the disorder, provide a rational on the use of MSC for the treatment of affected patients, as well as to briefly present and discuss pertinent research done in this context.

## 2 Main Text

### 2.1 Immune Dysregulation in ASD

It can be hypothesized that the development of subgroups of patients with ASD may follow a sequence of successive events, which include: 1) multiple stressful events during the critical neuro-developmental process, such as early infections or maternal autoantibodies interactions with specific immunogenetic background, 2) generation of immune dysfunction leading to further pro-inflammatory processes, gut- and blood-brain barriers alterations and autoimmune processes and 3) consequent brain alterations in otherwise genetically determined individuals.

#### 2.1.1 Susceptibility to Infections

Epidemiology has repeatedly shown an increased rate of ASD in children born from mothers who had an infection during pregnancy. These associations have been consistent across countries and time periods, and has been reported as early as the 1970s after the rubella pandemic in the United States (Chess, 1971) and throughout the decades in Denmark, Sweden and other countries (Atladóttir et al., 2010a). For instance, maternal viral infection requiring hospitalization during the first trimester or bacterial infections during the second trimester have been associated with a diagnosis of ASD in a large Danish cohort of 1,418,152 children of whom 7,379 had a diagnosis of ASD (Atladóttir et al., 2010b). Infections with intracellular pathogens were more frequently observed in patients with ASD; for example, Nicolson and al, reported a prevalence of 8.3% of infections with Chlamydia species in ASD subjects as compared to only 2.1% in healthy controls (Nicolson et al., 2007). A putative mechanism for a link with the neuronal activity was proposed by Vojdani who found an increased level of cross-reactive antibodies to Chlamydia determinants in patients with ASD (Vojdani et al., 2002). Infections with other intracellular pathogens such as Mycoplasma fermentans, Mycoplasma species (poly-infection), *Borrelia burgdorferi* (mono-infection or in combination with Rickettsia or Mycoplasma) have also been reported to be associated with ASD (Bransfield et al., 2008). The potential association of congenital rubella, herpes, cytomegalovirus, varicella, mumps, polyomavirus and enterovirus with ASD have also been cited in other reports (Stubbs, 1976; Stubbs, 1978; Libbey et al., 2005; Lintas et al., 2010). The occurrence of these infectious events might be mediated by genetic backgrounds that predisposes an individual to inappropriate response to environmental stressors.

Some genes associated with autism may reduce the capacity to respond to bacterial toxins, while other genetic factors may cause inappropriate/overwhelmed immune responses (Heuer et al., 2011; Onore et al., 2012). Associations with genes implicated in the immune response against pathogens, namely immunogenetics, may thus be related to the risk of developing ASD. For instance, a gene-gene expression analysis of brain samples of patients with ASD has identified two distinct gene expression modules associated with autism that might act in concert to disrupt normal brain development (Voineagu et al., 2011). Such observations indicate that the brain of patients with autism show altered expression of genes relating to normal synapses development and increased expression of genes that mediate inflammation. The genetic diversity of Dectin-1, which encodes a key molecule involved in fungal-mediated signaling in the gastro-intestinal tract, has been associated with ASD, suggesting a genetically determined inability to mount efficient innate immune responses against danger signals (Bennabi et al., 2015); similarly the MHC cluster through HLA alleles/haplotypes or complement C4 alleles either in patients (Odell et al., 2005; Lee et al., 2006; Johnson et al., 2009) or in their mothers (Heuer et al., 2011) has been reported to modify ASD risk.

#### 2.1.2 Pro-Inflammatory Processes

As a consequence of the above-mentioned triggering events along with inability to mount efficient anti-infectious responses, marked immune response in the brain and microglial cell activation is one of the most prominent ASD characteristics (Vargas et al., 2005; Li et al., 2009). In children diagnosed with ASD, brain tissue demonstrates excessive white matter growth regions along with inflammation (Vargas et al., 2005). In the peripheral blood, elevated circulating levels of pro-inflammatory cytokines such as interleukin (IL) IL-1beta, IL-6, IL-8, IL-12p40 (Jüttler et al., 2002; Ashwood et al., 2011) together with diminished anti-inflammatory cytokines (TGF-beta, IL10) (Enstrom et al., 2010; Ashwood et al., 2011) have been reported. The underlying mechanisms linking immune dysregulation and neuronal dysfunction are not clear, but there is evidence indicating that certain cytokines can impair neurodevelopment. IL-6, for example, has been described as a possible neuromodulator induced by neuronal activity regulating brain function, neurodevelopment (Jüttler et al., 2002). Elevated levels of IL-6 in the brain decrease the number of primary dendrites and have a negative impact in neuron survival and neuronal plasticity and, therby, potentially impacting behavior (Wei et al., 2013). Of interess, increased expression of IL-6 gene transcripts and IL-1 pathways, as well as increased circulating levels of IL-6 and IL-1 beta (Vargas et al., 2005; Li et al., 2009) have been noted in post mortem brains of patients with ASD.

#### 2.1.3 Immune Cells Abnormalities

Circulating monocytes from children with ASD display enhanced production of pro-inflammatory cytokines upon Toll-like receptor (TLR)-2 and TLR-4 dependent stimulation, while TLR-9 stimulation resulted in decreased cytokines production (Enstrom et al., 2010). Plasma levels of macrophage inhibitor factor (MIF), a pro-inflammatory immune regulator of both neural and endocrine systems, have been shown to be higher in ASD patients compared to healthy controls (Grigorenko et al., 2008). Further, several abnormal adaptive cellular responses have been reported such as altered T-cell activation (Ashwood et al., 2004), increased of basal levels of NK cells with diminished potential of response to stimulation (Enstrom et al., 2009), reduced number of CD4^+^ T cells, decreased of peripheral blood lymphocytes (Ashwood et al., 2003), inefficient response of T cell to mitogens (Molloy et al., 2006), and B cell dysfunction as evidenced by imbalance of serum immunoglobulin (Ig) levels (Enstrom et al., 2009). Bias towards T-helper2 (Th2) phenotype and reduced response of Th1 cells have also been documented (Noriega and Savelkoul, 2014). Plioplys et al. (1994) and Warren et al. (1992) demonstrated that a substantial number of subjects with autism have an increased number of HLA DR + activated T cells. Ashwood et al. (2004) found a higher number of B cells and NK cells in children with autism compared to controls as well as increased markers of cellular activation such as CD38 on B Cells, HLA-DR and CD26 on T cells subsets. Altogether, ASD patients display an increased activation of the innate and adaptive immune response.

#### 2.1.4 Autoimmune Processes

The association between auto-immunity and autism was first described in 1971 with the report of a child with autism and a family history of autoimmune diseases (Money et al., 1971) and further extended when families of children with autism were shown to have higher prevalence of autoimmune disorders (Comi et al., 1999; Atladóttir et al., 2010a). Croen et al. (2005), found that mothers who had developed autoimmune diseases during the second trimester of pregnancy carried a high risk of having a child with autism. It was reported that mothers of autistic patients had increased rates of rheumatoid arthritis (Atladóttir et al., 2010a). The presence of anti-fetal brain antibodies in the serum of mothers supports the hypothesis of an association between the maternal immune system and the diagnosis of ASD (Braunschweig et al., 2007; Braunschweig and Van de Water, 2012; Fox et al., 2012). In a different study, serum from a mother of an autistic child was found to bind to Purkinje cells and other neurons, and behavioral changes were induced in mice injected with these antibodies (Dalton et al., 2003). Though not yet tested in animal models, it is hypothesized that autoreactive antibodies in the maternal bloodstream can cross the placenta, enter the fetal circulation, and access the brain via a premature blood-brain barrier. The production of such auto-antibodies might be linked to a deregulated anti-infectious response, where molecular mimicry of antigenic components of the pathogen leads to cross-reactivity of antibodies. Another possibility is that unresolved infectious processes with repeated antigen stimulation may lead to chronic inflammation and autoimmunity with rupture of tolerance. Finally, we cannot exclude deregulated tolerogenic processes, which are mandatory for tolerance by the maternal immune system of the semi-allogeneic foetus at the fetal-placental interface (Ferreira et al., 2017). In this context, several studies have reported associations between molecular and genetic aspects of the potent immuno-regulatory HLA-G locus and ASD risk (Guerini et al., 2015; Guerini et al., 2018a; Guerini et al., 2018b; Guerini et al., 2019).

Various other auto-antibodies have been found in patients with autism: antibodies directed against 5HT receptors, myelin basic protein, neuron axon filament protein, cerebellar neurofilaments, nerve growth factor, alpha 2 adrenergic binding sites, and brain endothelial cell proteins (Ashwood et al., 2006). However, the pathogenic roles of such auto-antibodies are still unknown, and it is unclear whether they are a consequence or a cause of the disorder.

In summary, dysfunctions affecting the vast majority of immune processes are present from the early developmental stages through childhood and adulthood in subgroups of patients with ASD. Potential pathophysiologic mechanisms include alterations in maternal-fetal immune tolerance (via maternal antibodies and/or cellular immunity) and fetal brain inflammation, which may lead to changes in brain cytokines profile and microglial activation that may be detrimental for neurodevelopment. Evidence of increased numbers of microglia and increased microglial activation in ASD has been obtained via brain post-mortem studies, positron emission tomography (PET) brain imaging and animal models yielding offspring with ASD phenotypes by inducing immune activation in the pregnant women (Harvey and Boksa, 2012; Malkova et al., 2012).

The above-described immune dysfunctions, for example, genetically-determined non-resolved infectious events, even if resulting from a triggering event/process in the very early developmental stages, are still observed in adults with core ASD symptoms and reflected by elevated pro-inflammatory cytokines levels, highly activated T cells with consequent production of endogenous autoantibodies (Gesundheit et al., 2013; Gesundheit and Rosenzweig, 2017; Meltzer and Van de Water, 2017), suggesting a potential role of immune alterations and the associated chronic inflammation in the development or manifestation of ASD symptoms.

### 2.2 Microbiota Alteration and Intestinal Permeability

An increasing body of literature has reported that patients with ASD often have a combination of intestinal microbes that is distinct from healthy controls (Cao et al., 2013; De Angelis et al., 2013), and gastrointestinal symptoms are widely reported in patietents with ASD (Luna et al., 2017). Differences have been reported for the population of phyla *Bacteroidetes*, *Firmicutes* and genum *Clostridiales* among others (Finegold et al., 2010; Williams et al., 2011; Luna et al., 2017; Coretti et al., 2018). Convergent with these findings, one study found that Vancomycin, a powerful antibiotic targeting gram positive germs such as Clostridium, improved the symptoms in 8 out of 10 children with ASD and diarrhea (Sandler et al., 2000), but, a relapse of the symptoms relapsed shortly after antibiotic discontinuation.

Also, increased intestinal permeability (“leaky gut”) in patients with ASD, potentially related to abnormal microbiome, were observed in a study showing that 43% of children with ASD compared to 0% of the controls presented an abnormal mannitol:lactulose test (D'Eufemia et al., 19921996). Disrupted gut–blood–brain barriers may allow bacteria metabolites and other substances to get into the bloodstream and, subsequently, alter the brain (Al-Ayadhi et al., 2021).

The modified microbiota and the metabolites produced by the adapted intestinal microorganisms are likely to play an import role in the pathophysiology of ASD, particularly by having an effect in the production of neurotransmitters and interacting with the host nervous system, but also by impacting host immunity and inflammation (Xu et al., 2019).

All together, sustained abnormal microbiota and abnormal immune-inflammatory processes, increased oxydative stress induced by microbial, inflammatory and metabolic pathways could also explain the link between alterations in mitochondrial function and autism (Lombard, 1998; Pons et al., 2004).

In the context of ASD the potential role of cell therapy would be in attempting to address the immune dysfunction and inflammation that might develop in consequence of an altered microbiota.

### 2.3 Synaptic Dysfunction and Neuro-Structural Changes in ASD

Synapses are points of communication between neurons that the organized passage of information via electrical and chemical signaling. While there is a period of intensified synaptogenesis early in development, synapses retain plasticity throughout life, allowing the processes for learning and memory. Normal synaptic development and maintenance are essential to proper neuronal function, and abnormalities in either process have been associated with multiple neurodevelopmental conditions, including ASD. Additionally, human and animal studies have demonstrated a reduction in the size, number, and morphology of dendritic spines and an increase in immature spine morphology in ASD (Phillips and Pozzo-Miller, 2015). It is also likely that environmental factors influence synaptic changes. These alterations may lead to altered neuronal connectivity, such as large-range under-connectivity and short-range over-connectivity (Geschwind and Levitt, 2007; Maximo et al., 2014).

Individuals with ASD show structural and histological changes such as increased brain size, decreased number of Purkinje cells in the cerebellum and increased packing density with small body neurons in the hippocampus, the amygdala and the entorhinal cortex (Fatemi et al., 2012). These changes could indicate abnormal developmental processes involving neurogenesis, pruning and apoptosis. Connolly et al. (2006), unveiled the possibility that affected neurogenesis could be linked to immune processes in children with ASD by showing that abnormal neurogenesis could be mediated by increased levels of brain-derived neurotrophic factor (BDNF) and IgM/IgG anti-BDNF antibodies in sera of children with ASD when compared to neurotypical controls, even if the exact mechanism remains to be elucidated. Similar findings involving neurotrophic factors, such as nerve growth factor (NGF), were observed in a preliminary study in a Turkish population (Dinçel et al., 2013). Changes in the apoptotic process also seem important in ASD. Fatemi et al. (2001) describe decreased levels of anti-apoptotic Bcl-2 protein in ASD. A different study showed decreased anti-apoptotic signaling Bcl2 pathway paralleled by an increased expression of the pro-apoptotic p53 in the cerebellum of nine autistic patients (Sheikh et al., 2010). There is scarce neuroimaging study support to correlate the mitochondrial dysfunctions at the cerebral levels which, consequently, could explain changes in oxydative stress and energy production abnormalities. Yet, an older study using neuroimaging methods found that N-acetyl aspartate is decreased in the cerebellum of autistic children probably due to impaired mitochondrial functions (Chugani et al., 1999).

### 2.4 Mesenchymal Stromal Cells

MSCs, first identified by Friedenstein 45 years ago (Friedenstein et al., 1976), have since been extensively characterized. They can be isolated from the bone marrow, adipose tissue, umbilical cord blood, the placenta (Caplan and Dennis, 2006; Kern et al., 2006) and various other sources and can be relatively easily isolated and cultured *in vitro*. MSCs were defined in 2006 by the International Society of Cellular Therapy (ISCT) MSC committee as: 1) a plastic-adherent polyclonal population with fibroblast-like morphology, 2) positive for CD73, CD90 and CD105 markers (in >95% MSC), 3) negative for hematopoietic and endothelial markers, and 4) able to differentiate *in vitro* into osteoblasts, adipocytes, and chondroblasts (Dominici et al., 2006). MSCs primary mechanism of action is thought to result from immunomodulatory effects (Le Blanc and Davies, 2015; de Castro et al., 2019), mostly through their paracrine activities induced by inflammatory stimuli in the local milieu (Spees et al., 2016) (MSC priming), but also by cell-cell contact. MSCs interact with the innate and adaptative immune system on both the humoral and cell-mediated immune responses, including B-, T-, NK, dendritic-cell inhibition, decrease in pro-inflammatory cytokine production, and blocking neutrophil recruitment (Chamberlain et al., 2007; Gomez-Salazar et al., 2020).

Despite their ability to modulate the immune response, MSCs have long been considered as immuno-priviledged, promoting their use in the allogeneic setting across HLA barrier, due to their low level expression of MHC class I molecules on their cell surfaces and the lack of MHC class II expression and several co-stimulatory molecules at basal state. However, MSC exposure to inflammatory environnement increases the expression of MHC class I and induce MHC class II molecules, and the use of unmatched allogeneic MSC infusion may induce anti-HLA class I antibodies. Therefore, it is more appropriate to consider MSCs as “immune evasive” cells (Ankrum et al., 2014).

#### 2.4.1 Safety of MSCs

MSCs collected from the bone marrow (BM-MSC), umbilical cord (UC-MSC) and adipose tissue (AT-MSC) have been studied in multiple clinical trials worldwide involving thousands of individuals and a wide variety of human conditions. Their safety has been repeatedly demonstrated, and was summarized in a 2020 systematic review and meta-analysis of 55 clinical trials in 12 countries using MSCs in 2,700 recipients with various diseases. Based on randomized controlled trials, there was no association between MSC treatment and acute infusion-related toxicity, infection, thrombotic/embolic events, death, or malignancy (Thompson et al., 2020).

### 2.5 MSCs Immuno-Modulatory Properties: In Vitro Data

#### 2.5.1 MSCs and T Cells

MSCs obtained from healthy BM donors were initially shown to suppress *in vitro* T cells proliferation in a mixed lymphocyte reaction (MLR) (97). The inhibition has no immunological restriction as it was observed regardless of the MSCs origin: autologous or from a third party (Bartholomew et al., 2002; Tse et al., 2003). MSCs inhibit the proliferation of both naïve and memory CD4^+^ and CD8^+^ T cells through arrest in the G0/G1 phase of the cell cycle, and abrogate T cell activation (Glennie et al., 2005). In an inflammatory setting, MSCs appear to increase the number and activity of Treg cells and IL-10 expression, while suppressing Th1, Th2, and Th17 cells. MSC can also reduce the release of pro-inflammatory cytokines from different T cell populations, including interferon IFN-γ, TNF, IL-6, and IL-7, and increase the anti-inflammatory cytokines, such as IL-4 and IL- 10 (Aggarwal and Pittenger, 2005; Prevosto et al., 2007).

Typically, the mechanism of action of MSCs first involves the release of chemokines, allowing the attraction of activated T cells (Ren et al., 2008) that in turn prime MSCs towards an immunosuppressive phenotype, which then secrete several growth factors, cytokines, enzymes and hormones (e.g., VEGF, PDGF, ANG-1, IL-11, PGE2, TSG-6, SDF-1, HGF, IGF-1, and IDO) (Krampera et al., 2006; Polchert et al., 2008; Ren et al., 2008; Spees et al., 2016). MSCs secretion of the IFN-γ-inducible-indoleamine 2,3-dioxygenase (IDO) enzyme in human play a major role in inhibiting T lymphocytes proliferation via the degradation of tryptophan in metabolites (Ren et al., 2008; Menard et al., 2013). Other MSCs-secreted-soluble factors have also been proposed to contribute to the inhibition of T cell proliferation, including galectin-1 and 3, or IL-10 (Gieseke et al., 2007; Yang et al., 2009; Patel et al., 2010; Sioud et al., 2011). The capacity of MSCs to induce Treg is mediated by TGF-β and soluble HLA-G5, with IL-10, IL1Rα, and PGE2 release, which in turn interact with the Th17/Treg balance (Selmani et al., 2008; Terraza-Aguirre et al., 2020). MSCs also act through the release of MSC-derived exosomes, which content have immunosuppressive and immunomodulatory activity, and can favor Treg expansion (Zhang et al., 2018).

Direct cell to cell contact mechanisms are also part of the MSC immune effect on T cells, in particular throughout CD106/VCAM-1 and CD54/ICAM-1, that are both upregulated on MSC by TNFα, which play a critical role in MSC immunosuppressive capacities by favoring adhesion of MSC to T cells (Ren et al., 19502010). MSCs were also shown to directly promote apoptosis of activated T cells via the Fas/Fas ligand pathway (Akiyama et al., 2012) and to suppress T cell proliferation via engagement of the inhibitory molecule programmed death 1 (PD-1) by its ligands PD-L1 and PD-L2 (Augello et al., 2005).

#### 2.5.2 MSCs and B Cells

The interaction between MSCs and B cells is complex and depends on the culture environments and the type of cells (Ribeiro et al., 2013; Fan et al., 2016). MSCs were shown to inhibit B-cell proliferation, differenciation, antibody production, and chemotaxis under inflammatory conditions (Ribeiro et al., 2013; Fan et al., 2016). This inhibition involves MSC secretion of IFN-γ inducible IDO (Maby-El Hajjami et al., 2009) and appears to be indirect, since it requires the presence of CD4^+^ and CD8^+^ T cells (Rosado et al., 2015). Importantly, non-inflammatory resting MSCs do not inhibit B-cell proliferation but induce B-reg expansion.

#### 2.5.3 MSCs and NK Cells

MSCs and NK cells have been shown to interact *in vitro* and the outcome of this interaction may depend on the state of NK-cell activation and/or on the cytokines present in the milieu. MSCs are capable of inhibiting IL-2-induced proliferation of resting NK cells and partially inhibit the proliferation of activated NK cells and thereby the NK mediated cytotoxicity (Spaggiari et al., 2008). The effect of MSCs on NK cells depends both on cell contacts and on soluble factors synthesis. The secretion of PGE2 and HLA-G5 by MSCs are involved in the suppression of NK function (Spaggiari et al., 2008). Treatment of MSC with IFN-γ leads to the upregulation of MHC class I expression and the downregulation of NK cell receptor ULBP3 expression (Götherström et al., 2011), and thereby MSCs become more resistant to NK cell cytotoxicity (Krampera et al., 2006; Spaggiari et al., 2008; Götherström et al., 2011).

#### 2.5.4 MSCs and Myeloid Cells

MSCs immunomodulatory effect can also be explained by their ability to interfere with the differentiation, the maturation and the function of dendritic cells (DC) (Jiang et al., 2005; Ramasamy et al., 2007). Indeed, MSCs can inhibit antigen presentation by DC via negative regulation of the CD11c, CD83 and MHC Class II DC-cell surface molecules expression (Beyth et al., 2005). MSCs can halt the monocyte differentiation into DC, through secretion of PGE2 and IL-6 soluble factors (Nauta et al., 2006; Remes Lenicov et al., 2018).

MSCs also interact with macrophages with reciprocal immunosuppressive effects on both cell types. Direct cell contact between MSCs and pro-inflammatory M1-macrophages increases the MSCs immunosuppressive capacities by: 1) upregulating and enchiching CD54/ICAM-1 at the contact area on MSCs, which in turn favor the adhesion to T cells, and 2) increasing IDO expression (Espagnolle et al., 2017). MSCs effect on macrophages are mostly paracrine. IDO and PGE2 are involved in the polarization of monocytes into IL-10 secreting anti inflammatory M2 macrophages (Németh et al., 2009; Maggini et al., 2010; François et al., 2012).

Other immunosuppressive activities of MSC on myeloid cells are driven by soluble factors. These include CCL2 and CXCL12, which cooperate as a heterodimer to upregulate IL-10 in CCR2pos macrophages (Giri et al., 2020) and tumor necrosis factor-stimulated gene 6 (TSG-6), which promotes the early inhibition of neutrophil and macrophage activity at sites of inflammation and inhibits CXCL8-dependent neutrophil transendothelial migration and chemotaxis (Dyer et al., 19502014; Lee et al., 2009; Choi et al., 2011).

### 2.6 Relevant MSCs Pre-clinical and Clinical Trials

#### 2.6.1 MSCs in Neurologic Conditions

Numerous preclinical studies using MSCs infusion for diseases of the central nervous system suggest that MSCs can act through release of different neurotrophic, anti-inflammatory, and anti-apoptotic factors to promote recovery of the injured area and prevent further damage. Most studies were performed on adults with stroke, with a few additional reports on patients with neurodegenerative conditions or multiple sclerosis.

Several small studies using systemically administered autologous bone marrow-derived MSCs have been conducted in adults with acute or chronic stroke, with no significant side effects (Dulamea, 2015). A phase II study of an allogeneic MSC product (MultiStem) was recently conducted in 126 adults with stroke (65 treated, 61 placebo) (Mays and Deans, 2016). MSC therapy was well-tolerated. While there was no difference between placebo and treated patients in measures of stroke recovery, the treatment group had a lower rate of mortality and infections, associated with down regulation of inflammatory biomarkers including IL-6. In addition, patients who received MSCs earlier (24–36 h post-stroke vs. 36–48 h post-stroke) demonstrated more favorable recovery than patients who received later treatment or placebo.

A few clinical trials of autologous MSC therapy have been conducted in patients with multiple sclerosis. In a study including 25 patients with progressive multiple sclerosis treated with a single intrathecal autologous bone marrow-derived MSCs, the disease stabilized in half of the patients over a one-year period (Mohyeddin Bonab et al., 2012). Side effects, all transient and self-limited, included low-grade fever, nausea/vomiting, lower limb weakness, and headache, and were likely related to the intrathecal route of administration of MSCs. Another study that treated 10 patients, with progressive visual deficits due to multiple sclerosis, with a single intravenous dose of autologous bone marrow-derived MSC demonstrated improvements in visual acuity, visual evoked response latency, and optic nerve area (Karussis et al., 2010; Yamout et al., 2010).

A clinical trial has been conducted in seven patients with Parkinson’s disease who received a single dose of autologous bone marrow-derived MSCs injected into the sub-ventricular zone using stereotaxic surgery. Three patients demonstrated an improvement in disease symptoms with a follow-up of 10–36 months. Two additional patients reported subjective improvement of symptoms and reduction in drug dosage (Mendes Filho et al., 2018).

### 2.7 Cell Therapies in ASD

#### 2.7.1 Potential Mechanism of MSCs in Treating ASD

The exact mechanism of action of MSCs in ASD is the subject of ongoing investigations, but there are several potential means through which MSCs may exert therapeutic effects, including cell-mediated immunomodulation, molecular-mediated neuroprotection, and restoration of functional neurologic circuitry (Siniscalco et al., 2014). As ongoing immune dysregulation may contribute to the pathophysiology of subgroups of patients with ASD, suppressing the cell-mediated immune response with MSCs could have potential therapeutic benefit. MSCs may also provide neuroprotection through anti-inflammatory mechanisms by inhibiting neural apoptosis, microglial activation, astrocyte proliferation, and oxidative stress molecules (Gesundheit et al., 2015). Koh et al. (2015) demonstrated that UCB-derived MSCs promote neuron survival via secretion of neurotrophic factors. In several other models, MSCs were shown to have the ability to decrease both the number and the activation of microglial cells, which play a critical role in the development of ASD (Ooi et al., 2015; Jaimes et al., 2017). It is unclear if this phenomenon is caused by a direct effect of the MSCs or if it is mediated through activation of cytokines (i.e., TCP, IL-6). Finally, MSCs may also aid in synaptogenesis and restoration/regeneration of functional neurological pathways by supplying bioactive agents that stimulate the action of intrinsic neural progenitor cells. Various molecular targets have been implicated, including tissue plasminogen activator (tPA), synaptophysin, brain-derived neurotrophic factor (BDNF), and neurotransmitter receptors. Through a series of *in vitro* experiments via coculture, patch-clamp, inhibitory, and biochemical techniques, Koh et al. (2015) demonstrated that human UC- derived MSCs can induce synapse formation and enhance synaptic function and that thrombospondin proteins are both produced by UC-derived MSC and necessary for their synaptic effects.

The main dysregulations and other dysfunctions in ASD as well as the related potential mechanisms by which MSCs might aid in correcting or improving them are describe in Table 1.

**TABLE 1:** Observed immune and other dysregulations (peripheral, neurological and enteric) along with the MSC potential alleviating mechanisms.

Dysregulation	Potential mechanism of MSC
Immuno-genetically determined inability to mount efficient anti-infectious responses with consequent chronic inflammation	Overall restoration/improvement of the dysimmune pathways through;
Elevated pro-inflammatory cytokine levels	
Diminished anti-inflammatory cytokine levels	
Altered cytokine production by monocyte after TLR-based stimulation	
Altered CD8^+^ and CD4^+^ T-cell profiles	-Synthesis and releasing of anti-inflammatory cytokines and growth factors;
Altered NK cell activity	
Imbalance of serum immunoglobulin due to B cell dysfunction	
Peripheral and central autoimmune processes due to deregulated anti-infectious responses leading to inflammation and autoimmunity after rupture of tolerance and autoantibodies production	
Altered composition of intestinal microbiota leading to increased intestinal permeability (leaking of unwanted bacteria metabolites that might harm the brain) and chronic inflammation	-Suppression of cell-mediated immune response through inhibition of proliferation of several cell subsets including T-lymphocytes and NK cells
Altered immune-related transcriptome profiles	
Altered genome-wide expression profiles in lymphoblastoid cells	
Elevated activation of astrocytes and microglia	Provide neuroprotection through anti-inflammatory mechanisms by inhibiting microglial activation and astrocyte proliferation
Synaptic dysfunction and neuro-structural changes	Act in the existing neural and synaptic network to restore plasticity;
	Local secretion of substances that could reduce inflammation and promote tissue repair;
	Promote neuron survival via secretion of neurotrophic factors;
	Aid in synaptogenesis and regeneration of functional neurological pathways by supplying bioactive agents that stimulate the action of intrinsic neural progenitor cells

#### 2.7.2 Pre-Clinical Studies

Mouse models of single gene disorders that are associated with the ASD phenotype, such as Fragile X syndrome, Rett syndrome, and tuberous sclerosis complex syndrome, have been used to study the effects of cellular therapy on both brain and behavior. Reported data from such animal studies may reflect differences related to the specific genetic subtype of ASD and may not be generalized to all cases (e.g., idiopathic ASD). Derecki et al. (2012) report recovery of function in a mouse model of Rett syndrome, an X-linked condition associated with ASD typically caused by a mutation of the MECP2 gene. This gene encodes a methyl-CpG-binding protein, and the mutation leads to deficient phagocytic function in glial cells. Transplantation of cells from wild type bone marrow via intravenous infusion arrested disease development in the mouse model of Rett syndrome (Mecp2-null C57BL/6 mice). Following engraftment, survival was improved, breathing patterns normalized, apneas were reduced, body weight increased, and locomotor activity was improved. The BTBR T + Itpr3tfI (BTBR) mouse strain, derived from the inbred Black and Tan Brachyury strain, is another mouse model of ASD. In addition to impaired social behavior, aberrant communication, increased repetitive behaviors, and increased cognitive rigidity, BTBR mice also exhibit increased levels of peripheral CD4^+^ T-cells, peripheral B-cells, and serum and brain immunoglobulin levels, among other immune abnormalities was hence an interesting model for MSC-based treatment. Accordingly, Segal-Gavish et al. (2016) deliver human MSCs to BTBR mice via intraventricular injection into the central nervous system. Mice were immunosuppressed with cyclosporine before and after treatment. In this model, improvements in all three domains–social behavior, stereotyped behaviors, and cognitive rigidity–were observed in MSC-treated mice compared to controls. However, differences in anxiety-related behaviors and locomotion were not observed.

In two separate studies, Perets et al. (2018) showed that intranasal administration of purified exosomes derived from mesenchymal stem cell improved autistic like behaviors such as social interactions and vocalization, and decreased repetitive behavior in BTBR and Shank3B mice, respectively (Perets et al., 2020).

These murine models were all favorable arguments to test the potential benefit of cellular therapies, in subtypes of ASD.

#### 2.7.3 Clinical Trials of Cell Therapies in ASD-Clinical Trial Registries

Several clinical trials of cell therapies for ASD have been conducted or proposed. A search performed on *ClinicalTrials.gov (May 2021)* using the terms “(Autism Spectrum Disorder OR Autism OR Autistic Spectrum Disorder | Cell Therapy OR Cellular Therapy.”) yield 43 studies, of which only 18 actually turned out to have cell therapies interventions for ASD. Two of the studies had a “withdrawn” status. Form the remaining 16 studies, only 3 involved MSC infusions. No trials involving MSCs and autism were identified in the European Clinical Trial Registry.

In a review of cell therapies for ASD recently published, Price et all present the results of 13 studies (2 on MSCs) that they identified with a similar search in December of 2019. In their review, the authors discuss the published results and conclude that the data available on cell therapy for ASD is still very limited and does not allow for comparison among the different studies. Nevertheless, the authors firmly state that the studies reviewed consistently demonstrated the safety of cell therapy interventions and that from this point forward only placebo controlled studies are justifiable (Price, 2020).

#### 2.7.4 Clinical Trials of Cell Therapies in ASD-From UCB to Umbilical Cord-Derived MSCs

The collection of MSCs from UC blood or tissue is non-invasive, easier, and less expensive than the collection from other tissue types. Moreover, UC-derived cells are potentially less immunogenic due to their immaturity (Divya et al., 2012).

Although MSCs retain their inducible immunoregulatory properties regardless of the tissue type they derive from (Amati et al., 2017), it is now recognized that some of their biological properties vary, with consequent potential repercussion in their clinical applications (Marquez-Curtis et al., 2015; Liu et al., 2016). In addition, a previous study suggested that the expanded UC-derived MSCs include a small unique population of cells that express both MSC and pluripotent stem cell markers resulting in an innate neurogenic potential (Divya et al., 2012).

Also, it has been demonstrated that effector cells present in umbilical cord blood are able to alter brain connectivity through paracrine signaling and also to control inflammation (Bachstetter et al., 2008; Shahaduzzaman et al., 2013; Dawson et al., 2017).

Investigators from Duke University in the United States completed an open-label phase 1, safety and tolerability, study in 25 children diagnosed with ASD who were treated with autologous UCB and followed for a year (NCT02176317). UCB was administered as a single infusion (median infused dose of 2.6 × 10^7^/kg) with no prior immunosuppression. The safety and tolerability profile of autologous UCB infusion in ASD was excellent. Improvement in social communication abilities were noted on the caregiver-completed Vineland Adaptive Behavior Scales-Second Edition (VABS-II) and on the Pervasive Developmental Disorder Behavior Inventory (PDDBI). The Clinical Global Impression- Improvement scale, completed by clinicians, reflected beneficial changes during the 6- month period post-infusion in core ASD symptoms in approximately 60% of the participants, as manifested by the participants increased social communication skills, receptive/expressive language, decreased repetitive behavior, and decreased sensory sensitivities (Dawson et al., 2017). In a secondary analysis, researchers showed that electrophysiological biomarkers could predict improvement in autistic symptoms (Murias et al., 2018) and that behavioral and social communication improvements were associated with specific changes in the brain (Carpenter et al., 2019; Simhal et al., 2020). The same group proceeded with a phase 2 double-blind randomized study (NCT02847182) to evaluate the safety and efficacy of UCB, compared to placebo, in improving social communication abilities in 180 children with ASD. The children enrolled in the trial received a single intravenous autologous (*n* = 56) or allogeneic (*n* = 63) UCB infusion, or a placebo (*n* = 61). The infusions were well tolerated and patients were evaluated 6 months after the procedure. The results showed no evidence of improvement in social communication or other autism symptoms. However, in a subgroup of children without intellectual deficit, significant improvement in communication skills, exploratory measures (attention to toys and sustained attention), and increased alpha and beta electroencephalographic power were observed in those treated with UCB (Dawson et al., 2020).

A similar phase II clinical trial (NCT01638819) including 29 children with ASD who received autologous UCB infusion performed by a different group of researchers also in the United States Although a trend for socialization improvement was observed, the results were not statistically significant (Chez et al., 2018).

After the clinical trials with UCB therapy in patients with ASD, the Duke University group completed an open-label, phase I study (NCT03099239) on 12 children treated with umbilical cord (UC) derived MSCs from an unrelated donor. The enrolled children received 1, 2 or 3 doses of 2 × 10^6^/Kg in intervals of 2 months, respectively, and were assessed at baseline, 6 months and 1 year after infusion. The infusions were well tolerated except for some reports of agitation during the procedure. Class I HLA antibodies, without any currently know clinically significance, were detected in 5 of the treated patients. Half of the patients included in the trial showed improvement in autistic symptoms in at least two of the specific measures considered, but the observed improvements could not be attributed with certainty to the treatment. The clinical trial confirmed the safety of the use of MSCs for treating children with autism, but the efficacy of the treatment still warrants further evaluation (Sun et al., 2020).

In addition, the same research group has two ongoing clinical trials to investigate the use of UC- derived MSCs in pediatric patients with ASD: 1) the TACT, an open label phase I trial (NCT04294290) to evaluate the safety and feasibility of the treatment in toddlers aged 18–48 months; 2) the IMPACT (NCT04089579) which is a randomized, double blinded, phase II study to determine the efficacy UC -derived MSCs in children with ASD aged 4–11 years. Results for the trials are expected by the end of 2022 and mid-2023, respectively. They have also proposed an open label, phase I trial to evaluate the safety and feasibility of the use of UC derived MSCs in adults with ASD, but at the time this review was written, patient recruitment had not yet started.

A summary of the publications associated with the clinical trials on ASD using UCB or MSCs in patients with ASD (Lv et al., 2013; Dawson et al., 2017; Chez et al., 2018; Murias et al., 2018; Carpenter et al., 2019; Simhal et al., 2020; Sun et al., 2020), including those detailed above, are provided in Table 2.

**TABLE 2:** Summary of Publications Issued from Clinical Trials on ASD Using MSC or UCB.

Study number*	Title	Study characteristics/	Cell type	Population	Main findings	Authorship	Journal
NCT01343511	Transplantation of human cord blood mononuclear cells and umbilical cord-derived mesenchymal stem cells in autism	Phase I/II, open-label clinical trial	CBMNC and UC-derived MSCs	37 children with ASD (CBMNC *n* = 14, CBMNCs and MSCs combined *n* = 9, control *n* = 14); median age of 7.08 years (range 6.51–5.02)	Treatment was deemed safe and well tolerated	LV et al. (2013)	J Transl Med
					Infusion of CBMNCs and MSCs combined showed more effects than the CBMNC transplantation alone and control		
NCT02176317	Autologous cord blood infusions are safe and feasible in young children with autism spectrum disorder: Results of a single-center phase I open-label trial	Phase I, open-label clinical trial	Autologous UCB	25 children with ASD; 72% with moderately severe or severe symptoms; median age of 4.62 years (range 2.26–5.97)	Treatment was deemed safe and well tolerated; only mild and moderated AE reported. Improvements of ASD symptoms were observed within the 6 months after infusion.	Dawson et al. (2017)	Transl Med
	White matter tract changes associated with clinical improvement in an open-label trial assessing autologous umbilical cord blood for treatment of young children with autism	Study conducted as part of NCT02176317			Behavioral improvements after UCB infusion were associated with increased white matter connectivity.	Carpenter et al. (2019)	Stem Cells Transl Med
	Measuring robustness of brain networks in autism spectrum disorder with Ricci curvature	Study conducted as part of NCT02176317; secondary analysis using diffusion tensor imaging (DTI) data.			The study showed that Ricci curvature identifies changes in robustness in brain regions that are correlated with improvements in social communication; these changes were not detected via traditional brain network analysis	Simhal et al. (2020)	Sci Rep
	Electrophysiological biomarkers predict clinical improvement in an open-label trial assessing efficacy of autologous umbilical cord blood for treatment of autism	Study conducted as part of NCT02176317			Significant changes in spectral characteristics observed by 12 months post-infusion; association of higher baseline posterior EEG beta power with a greater degree of improvement in social communication symptoms	Murias et al. (2018)	Stem Cells Transl Med
NCT01638819	Safety and observations from a placebo‐controlled, crossover study to assess use of autologous umbilical cord blood stem cells to improve symptoms in children with autism	Phase II, randomized, blinded, placebo‐controlled, crossover trial	Autologous UCB	29 children with ASD; mean age of 4.53 years (range 2.42–6.80)	Treatment was safe no serious adverse events reported; trend towards improvement in socialization (not statistically significant)	Chez et al. (2018)	Stem Cells Transl Med
NCT03099239	Infusion of human umbilical cord tissue mesenchymal stromal cells in children with autism spectrum disorder.	Phase I, open-label.	Allogeneic UC-derived MSC	12 children with ASD; -median age of 6.4 years (range 4–9)	Treatment was deemed safe, feasible, and well tolerated, except for agitation during procedure; 5 patients developed anti-HLA antibodies, but with no clinical manifestations; -Half of the participants showed improvement in at least 2 ASD measures	Sun et al. (2020)	Stem Cells Transl Med
NCT02847182	A phase II randomized clinical trial of the safety and efficacy of intravenous umbilical cord blood infusion for treatment of children with autism spectrum disorder	Phase II, prospective randomized, placebo-controlled, double-blind	Autologous or allogeneic UCB	180 children with ASD, aged 2–7 years (mean 5.47); autologous UCB (*n* = 56), allogeneic UCB (*n* = 63), placebo (*n* = 61)	UCB infusion was, in general, not associated with decrease in ASD symptoms or improvements in socialization; in a subgroup of children without intellectual disability improvements in communication skills was observed	Dawson et al. (2020)	The Journal of Pediatrics

## 3 Conclusion and Further Perspective

The immune-brain axis is now well known to play a primordial key role in the development of ASD as evidenced by the existence of inefficient anti-infectious responses, low grade inflammation, immune-cell subset alterations and autoimmunity. All these immune dysfunctions are similar to those observed in common inflammatory/autoimmune chronic disorders, known to be improved by stem cell-based therapies, including those using MSCs. Correcting or improving the immune dysfunctions in patients with ASD, via direct or indirect mechanisms, might induce changes that would potentially result in improvement in core domains of autistic symptomatology in these patients and contribute to increasing their quality of life. Thus, continuing research in this field is encouraged, in particular future studies considering cohorts selected on the basis of immune dysfunction parameters that seem essential to determine the efficacy of MSC in this context.

The mechanistic rationale and overarching theory of this line of investigation is that MSCs can act through paracrine and allocrine mechanisms to modulate ongoing inflammation and/or immune pathology in the brain and possibly protect neurons from further damage. In many contexts, MSCs dampen, rather than augment/overwhelm, immunological and inflammatory responses. Documented mechanisms include shifts in effector T cells such as generation of regulatory T cell populations and changes in monocyte/dendritic cell cytokine generation leading to anti-inflammatory cytokines. Therefore, it is plausible to consider a population of MSCs as an immunological and/or anti-inflammatory agent. Both post-mortem brain tissue studies and PET imaging data from living individuals with ASD have revealed evidence of increased microglial activation, suggesting that immune and/or inflammatory mediated brain damage plays a role in the etiology of ASD as discussed above.

The combination of ASD symptoms and the related co-morbidities places a very high burden on the affected individual, their families and society. Because of the lack of effective treatments, non-evidence-based interventions, often costly, are usually sought by concerned families, making ASD, an area in crucial need for therapeutic innovation. Developing drugs for ASD has been challenging because of a limited understanding of its underlying pathophysiology. In this frustrating context, emerging evidences shed light towards a primordial key role of immune dysfunction in ASD characterized by inflammation, T-lymphocyte abnormalities, autoantibodies resulting in structural brain changes and abnormal immune mediation of synaptic functions, which serve as the rationale for immune-related interventions such as MSC-based treatment. Successful treatments will reduce care costs, increase productivity and family income and increase family quality of life.

## References

[B1] Abrahams B. S. Geschwind D. H. (2010). Connecting Genes to Brain in the Autism Spectrum Disorders. Arch. Neurol. 67, 395–399. 10.1001/archneurol.2010.47 PubMed Abstract | 10.1001/archneurol.2010.47 | Google Scholar 20385903 PMC3645845

[B2] Aggarwal S. Pittenger M. F. (2005). Human Mesenchymal Stem Cells Modulate Allogeneic Immune Cell Responses. Blood 105, 1815–1822. 10.1182/blood-2004-04-1559 PubMed Abstract | 10.1182/blood-2004-04-1559 | Google Scholar 15494428

[B3] Akiyama K. Chen C. Wang D. Xu X. Qu C. Yamaza T. (2012). Mesenchymal-stem-cell-induced Immunoregulation Involves FAS-Ligand-/fas-Mediated T Cell Apoptosis. Cell Stem Cell 10, 544–555. 10.1016/j.stem.2012.03.007 PubMed Abstract | 10.1016/j.stem.2012.03.007 | Google Scholar 22542159 PMC3348385

[B4] Al-Ayadhi L. Zayed N. Bhat R. S. Moubayed N. M. S. Al-Muammar M. N. El-Ansary A. (2021). The Use of Biomarkers Associated with Leaky Gut as a Diagnostic Tool for Early Intervention in Autism Spectrum Disorder: a Systematic Review. Gut Pathog. 13, 54. 10.1186/s13099-021-00448-y PubMed Abstract | 10.1186/s13099-021-00448-y | Google Scholar 34517895 PMC8439029

[B5] Amati E. Sella S. Perbellini O. Alghisi A. Bernardi M. Chieregato K. (2017). Generation of Mesenchymal Stromal Cells from Cord Blood: Evaluation of *In Vitro* Quality Parameters Prior to Clinical Use. Stem Cell Res. Ther. 8, 14. 10.1186/s13287-016-0465-2 PubMed Abstract | 10.1186/s13287-016-0465-2 | Google Scholar 28115021 PMC5260040

[B6] American Psychiatric Association (2013). Diagnostic and Statistical Manual of Mental Disorders. Fifth Edition. American Psychiatric Association. 10.1176/appi.books.9780890425596 10.1176/appi.books.9780890425596 | Google Scholar

[B7] Ankrum J. A. Ong J. F. Karp J. M. (2014). Mesenchymal Stem Cells: Immune Evasive, Not Immune Privileged. Nat. Biotechnol. 32, 252–260. 10.1038/nbt.2816 PubMed Abstract | 10.1038/nbt.2816 | Google Scholar 24561556 PMC4320647

[B8] Ashwood P. Anthony A. Torrente F. Wakefield A. J. (2004). Spontaneous Mucosal Lymphocyte Cytokine Profiles in Children with Autism and Gastrointestinal Symptoms: Mucosal Immune Activation and Reduced Counter Regulatory Interleukin-10. J. Clin. Immunol. 24, 664–673. 10.1007/s10875-004-6241-6 PubMed Abstract | 10.1007/s10875-004-6241-6 | Google Scholar 15622451

[B9] Ashwood P. Krakowiak P. Hertz-Picciotto I. Hansen R. Pessah I. Van de Water J. (2011). Elevated Plasma Cytokines in Autism Spectrum Disorders Provide Evidence of Immune Dysfunction and Are Associated with Impaired Behavioral Outcome. Brain, Behav. Immun. 25, 40–45. 10.1016/j.bbi.2010.08.003 10.1016/j.bbi.2010.08.003 | Google Scholar 20705131 PMC2991432

[B10] Ashwood P. Murch S. H. Anthony A. Pellicer A. A. Torrente F. Thomson M. A. (2003). Intestinal Lymphocyte Populations in Children with Regressive Autism: Evidence for Extensive Mucosal Immunopathology. J. Clin. Immunol. 23, 504–517. 10.1023/b:joci.0000010427.05143.bb PubMed Abstract | 10.1023/b:joci.0000010427.05143.bb | Google Scholar 15031638

[B11] Ashwood P. Wills S. Van de Water J. (2006). The Immune Response in Autism: a New Frontier for Autism Research. J. Leukoc. Biol. 80, 1–15. 10.1189/jlb.1205707 PubMed Abstract | 10.1189/jlb.1205707 | Google Scholar 16698940

[B12] Atladóttir H. Ó. Thorsen P. Østergaard L. Schendel D. E. Lemcke S. Abdallah M. (2010). Maternal Infection Requiring Hospitalization during Pregnancy and Autism Spectrum Disorders. J. Autism Dev. Disord. 40, 1423–1430. 10.1007/s10803-010-1006-y PubMed Abstract | 10.1007/s10803-010-1006-y | Google Scholar 20414802

[B13] Atladóttir H. Ó. Thorsen P. Schendel D. E. Østergaard L. Lemcke S. Parner E. T. (2010). Association of Hospitalization for Infection in Childhood with Diagnosis of Autism Spectrum Disorders: a Danish Cohort Study. Arch. Pediatr. Adolesc. Med. 164, 470–477. 10.1001/archpediatrics.2010.9 PubMed Abstract | 10.1001/archpediatrics.2010.9 | Google Scholar 20439799

[B14] Augello A. Tasso R. Negrini S. M. Amateis A. Indiveri F. Cancedda R. (2005). Bone Marrow Mesenchymal Progenitor Cells Inhibit Lymphocyte Proliferation by Activation of the Programmed Death 1 Pathway. Eur. J. Immunol. 35, 1482–1490. 10.1002/eji.200425405 PubMed Abstract | 10.1002/eji.200425405 | Google Scholar 15827960

[B15] Bachstetter A. D. Pabon M. M. Cole M. J. Hudson C. E. Sanberg P. R. Willing A. E. (2008). Peripheral Injection of Human Umbilical Cord Blood Stimulates Neurogenesis in the Aged Rat Brain. BMC Neurosci. 9, 22. 10.1186/1471-2202-9-22 PubMed Abstract | 10.1186/1471-2202-9-22 | Google Scholar 18275610 PMC2268935

[B16] Bartholomew A. Sturgeon C. Siatskas M. Ferrer K. McIntosh K. Patil S. (2002). Mesenchymal Stem Cells Suppress Lymphocyte Proliferation *In Vitro* and Prolong Skin Graft Survival *In Vivo* . Exp. Hematol. 30, 42–48. 10.1016/s0301-472x(01)00769-x PubMed Abstract | 10.1016/s0301-472x(01)00769-x | Google Scholar 11823036

[B17] Bennabi M. Delorme R. Oliveira J. Fortier C. Lajnef M. Boukouaci W. (2015). Dectin-1 Polymorphism: A Genetic Disease Specifier in Autism Spectrum Disorders? PloS One 10, e0137339. 10.1371/journal.pone.0137339 PubMed Abstract | 10.1371/journal.pone.0137339 | Google Scholar 26352598 PMC4564239

[B18] Bennabi M. Gaman A. Delorme R. Boukouaci W. Manier C. Scheid I. (2018). HLA-class II Haplotypes and Autism Spectrum Disorders. Sci. Rep. 8, 7639. 10.1038/s41598-018-25974-9 PubMed Abstract | 10.1038/s41598-018-25974-9 | Google Scholar 29769579 PMC5955937

[B19] Beyth S. Borovsky Z. Mevorach D. Liebergall M. Gazit Z. Aslan H. (2005). Human Mesenchymal Stem Cells Alter Antigen-Presenting Cell Maturation and Induce T-Cell Unresponsiveness. Blood 105, 2214–2219. 10.1182/blood-2004-07-2921 PubMed Abstract | 10.1182/blood-2004-07-2921 | Google Scholar 15514012

[B20] Bransfield R. C. Wulfman J. S. Harvey W. T. Usman A. I. (2008). The Association between Tick-Borne Infections, Lyme Borreliosis and Autism Spectrum Disorders. Med. Hypotheses 70, 967–974. 10.1016/j.mehy.2007.09.006 PubMed Abstract | 10.1016/j.mehy.2007.09.006 | Google Scholar 17980971

[B21] Braunschweig D. Ashwood P. Krakowiak P. Hertzpicciotto I. Hansen R. Croen L. (2007). Autism: Maternally Derived Antibodies Specific for Fetal Brain Proteins. Neurotoxicology 29, 226–231. 10.1016/j.neuro.2007.10.010 PubMed Abstract | 10.1016/j.neuro.2007.10.010 | Google Scholar 18078998 PMC2305723

[B22] Braunschweig D. Van de Water J. (2012). Maternal Autoantibodies in Autism. Arch. Neurol. 69, 693–699. 10.1001/archneurol.2011.2506 PubMed Abstract | 10.1001/archneurol.2011.2506 | Google Scholar 22689191 PMC4776649

[B23] Brown A. S. Cheslack-Postava K. Rantakokko P. Kiviranta H. Hinkka-Yli-Salomäki S. McKeague I. W. (2018). Association of Maternal Insecticide Levels with Autism in Offspring from a National Birth Cohort. Ajp 175, 1094–1101. 10.1176/appi.ajp.2018.17101129 PubMed Abstract | 10.1176/appi.ajp.2018.17101129 | Google Scholar PMC6377859 30111184

[B24] Buescher A. V. S. Cidav Z. Knapp M. Mandell D. S. (2014). Costs of Autism Spectrum Disorders in the United Kingdom and the United States. JAMA Pediatr. 168, 721–728. 10.1001/jamapediatrics.2014.210 PubMed Abstract | 10.1001/jamapediatrics.2014.210 | Google Scholar 24911948

[B25] Cao X. Lin P. Jiang P. Li C. (2013). Characteristics of the Gastrointestinal Microbiome in Children with Autism Spectrum Disorder: a Systematic Review. Shanghai Arch. Psychiatry 25, 342–353. 10.3969/j.issn.1002-0829.2013.06.003 PubMed Abstract | 10.3969/j.issn.1002-0829.2013.06.003 | Google Scholar 24991177 PMC4054584

[B26] Caplan A. I. Dennis J. E. (2006). Mesenchymal Stem Cells as Trophic Mediators. J. Cell. Biochem. 98, 1076–1084. 10.1002/jcb.20886 PubMed Abstract | 10.1002/jcb.20886 | Google Scholar 16619257

[B27] Carpenter K. L. H. Major S. Tallman C. Chen L. W. Franz L. Sun J. (2019). White Matter Tract Changes Associated with Clinical Improvement in an Open-Label Trial Assessing Autologous Umbilical Cord Blood for Treatment of Young Children with Autism. Stem Cells Transl. Med. 8, 138–147. 10.1002/sctm.18-0251 PubMed Abstract | 10.1002/sctm.18-0251 | Google Scholar 30620122 PMC6344899

[B28] Chamberlain G. Fox J. Ashton B. Middleton J. (2007). Concise Review: Mesenchymal Stem Cells: Their Phenotype, Differentiation Capacity, Immunological Features, and Potential for Homing. Stem Cells Dayt Ohio 25, 2739–2749. 10.1634/stemcells.2007-0197 10.1634/stemcells.2007-0197 | Google Scholar 17656645

[B29] Chaste P. Leboyer M. (2012). Autism Risk Factors: Genes, Environment, and Gene-Environment Interactions. Dialogues Clin. Neurosci. 14, 281–292. 10.31887/dcns.2012.14.3/pchaste PubMed Abstract | 10.31887/dcns.2012.14.3/pchaste | Google Scholar 23226953 PMC3513682

[B30] Chess S. (1971). Autism in Children with Congenital Rubella. J. Autism Dev. Disord. 1, 33–47. 10.1007/BF01537741 PubMed Abstract | 10.1007/BF01537741 | Google Scholar 5172438

[B31] Chez M. Lepage C. Parise C. Dang-Chu A. Hankins A. Carroll M. (2018). Safety and Observations from a Placebo-Controlled, Crossover Study to Assess Use of Autologous Umbilical Cord Blood Stem Cells to Improve Symptoms in Children with Autism. Stem Cells Transl. Med. 7, 333–341. 10.1002/sctm.17-0042 PubMed Abstract | 10.1002/sctm.17-0042 | Google Scholar 29405603 PMC5866927

[B32] Choi H. Lee R. H. Bazhanov N. Oh J. Y. Prockop D. J. (2011). Anti-inflammatory Protein TSG-6 Secreted by Activated MSCs Attenuates Zymosan-Induced Mouse Peritonitis by Decreasing TLR2/NF-Κb Signaling in Resident Macrophages. Blood 118, 330–338. 10.1182/blood-2010-12-327353 PubMed Abstract | 10.1182/blood-2010-12-327353 | Google Scholar 21551236 PMC3138686

[B33] Chugani D. C. Sundram B. S. Behen M. Lee M.-L. Moore G. J. (1999). Evidence of Altered Energy Metabolism in Autistic Children. Prog. Neuro-Psychopharmacology Biol. Psychiatry 23, 635–641. 10.1016/s0278-5846(99)00022-6 10.1016/s0278-5846(99)00022-6 | Google Scholar 10390722

[B34] Comi A. M. Zimmerman A. W. Frye V. H. Law P. A. Peeden J. N. (1999). Familial Clustering of Autoimmune Disorders and Evaluation of Medical Risk Factors in Autism. J. Child. Neurol. 14, 388–394. 10.1177/088307389901400608 PubMed Abstract | 10.1177/088307389901400608 | Google Scholar 10385847

[B35] Connolly A. M. Chez M. Streif E. M. Keeling R. M. Golumbek P. T. Kwon J. M. (2006). Brain-derived Neurotrophic Factor and Autoantibodies to Neural Antigens in Sera of Children with Autistic Spectrum Disorders, Landau-Kleffner Syndrome, and Epilepsy. Biol. Psychiatry 59, 354–363. 10.1016/j.biopsych.2005.07.004 PubMed Abstract | 10.1016/j.biopsych.2005.07.004 | Google Scholar 16181614

[B36] Corcione A. Benvenuto F. Ferretti E. Giunti D. Cappiello V. Cazzanti F. (2006). Human Mesenchymal Stem Cells Modulate B-Cell Functions. Blood 107, 367–372. 10.1182/blood-2005-07-2657 PubMed Abstract | 10.1182/blood-2005-07-2657 | Google Scholar 16141348

[B37] Corcoran J. Berry A. Hill S. (2015). The Lived Experience of US Parents of Children with Autism Spectrum Disorders: a Systematic Review and Meta-Synthesis. J. Intellect. Disabil. 19, 356–366. 10.1177/1744629515577876 PubMed Abstract | 10.1177/1744629515577876 | Google Scholar 25819433

[B38] Coretti L. Paparo L. Riccio M. P. Amato F. Cuomo M. Natale A. (2018). Gut Microbiota Features in Young Children with Autism Spectrum Disorders. Front. Microbiol. 9, 3146. 10.3389/fmicb.2018.03146 PubMed Abstract | 10.3389/fmicb.2018.03146 | Google Scholar 30619212 PMC6305749

[B39] Croen L. A. Grether J. K. Yoshida C. K. Odouli R. Van de Water J. (2005). Maternal Autoimmune Diseases, Asthma and Allergies, and Childhood Autism Spectrum Disorders: a Case-Control Study. Arch. Pediatr. Adolesc. Med. 159, 151–157. 10.1001/archpedi.159.2.151 PubMed Abstract | 10.1001/archpedi.159.2.151 | Google Scholar 15699309

[B40] D'Eufemia P. Celli M. Finocchiaro R. Pacifico L. Viozzi L. Zaccagnini M. (19921996). Abnormal Intestinal Permeability in Children with Autism. Acta Paediatr. Oslo Nor. 85, 1076–1079. 10.1111/j.1651-2227.1996.tb14220.x 10.1111/j.1651-2227.1996.tb14220.x | Google Scholar 8888921

[B41] Dalton P. Deacon R. Blamire A. Pike M. McKinlay I. Stein J. (2003). Maternal Neuronal Antibodies Associated with Autism and a Language Disorder. Ann. Neurol. 53, 533–537. 10.1002/ana.10557 PubMed Abstract | 10.1002/ana.10557 | Google Scholar 12666123

[B42] Dawson G. Sun J. M. Baker J. Carpenter K. Compton S. Deaver M. (2020). A Phase II Randomized Clinical Trial of the Safety and Efficacy of Intravenous Umbilical Cord Blood Infusion for Treatment of Children with Autism Spectrum Disorder. J. Pediatr. 222, 164–173. 10.1016/j.jpeds.2020.03.011 PubMed Abstract | 10.1016/j.jpeds.2020.03.011 | Google Scholar 32444220

[B43] Dawson G. Sun J. M. Davlantis K. S. Murias M. Franz L. Troy J. (2017). Autologous Cord Blood Infusions Are Safe and Feasible in Young Children with Autism Spectrum Disorder: Results of a Single-Center Phase I Open-Label Trial. Stem Cells Transl. Med. 6, 1332–1339. 10.1002/sctm.16-0474 PubMed Abstract | 10.1002/sctm.16-0474 | Google Scholar 28378499 PMC5442708

[B44] De Angelis M. Piccolo M. Vannini L. Siragusa S. De Giacomo A. Serrazzanetti D. I. (2013). Fecal Microbiota and Metabolome of Children with Autism and Pervasive Developmental Disorder Not Otherwise Specified. PloS One 8, e76993. 10.1371/journal.pone.0076993 PubMed Abstract | 10.1371/journal.pone.0076993 | Google Scholar 24130822 PMC3793965

[B45] de Castro L. L. Lopes-Pacheco M. Weiss D. J. Cruz F. F. Rocco P. R. M. (2019). Current Understanding of the Immunosuppressive Properties of Mesenchymal Stromal Cells. J. Mol. Med. 97, 605–618. 10.1007/s00109-019-01776-y PubMed Abstract | 10.1007/s00109-019-01776-y | Google Scholar 30903229

[B46] Derecki N. C. Cronk J. C. Lu Z. Xu E. Abbott S. B. G. Guyenet P. G. (2012). Wild-type Microglia Arrest Pathology in a Mouse Model of Rett Syndrome. Nature 484, 105–109. 10.1038/nature10907 PubMed Abstract | 10.1038/nature10907 | Google Scholar 22425995 PMC3321067

[B47] Di Nicola M. Carlo-Stella C. Magni M. Milanesi M. Longoni P. D. Matteucci P. (2002). Human Bone Marrow Stromal Cells Suppress T-Lymphocyte Proliferation Induced by Cellular or Nonspecific Mitogenic Stimuli. Blood 99, 3838–3843. 10.1182/blood.V99.10.3838 PubMed Abstract | 10.1182/blood.V99.10.3838 | Google Scholar 11986244

[B48] Dinçel N. Ünalp A. Kutlu A. Öztürk A. Uran N. Ulusoy S. (2013). Serum Nerve Growth Factor Levels in Autistic Children in Turkish Population: a Preliminary Study. Indian J. Med. Res. 138. Available at: https://pubmed.ncbi.nlm.nih.gov/24521633/ . Google Scholar PMC3978979 24521633

[B49] Divya M. S. Roshin G. E. Divya T. S. Rasheed V. A. Santhoshkumar T. R. Elizabeth K. E. (2012). Umbilical Cord Blood-Derived Mesenchymal Stem Cells Consist of a Unique Population of Progenitors Co-expressing Mesenchymal Stem Cell and Neuronal Markers Capable of Instantaneous Neuronal Differentiation. Stem Cell Res. Ther. 3, 57. 10.1186/scrt148 PubMed Abstract | 10.1186/scrt148 | Google Scholar 23253356 PMC3580487

[B50] Dominici M. Le Blanc K. Mueller I. Slaper-Cortenbach I. Marini F. C. Krause D. S. (2006). Minimal Criteria for Defining Multipotent Mesenchymal Stromal Cells. The International Society for Cellular Therapy Position Statement. Cytotherapy 8, 315–317. 10.1080/14653240600855905 PubMed Abstract | 10.1080/14653240600855905 | Google Scholar 16923606

[B51] Dulamea A. O. (2015). The Potential Use of Mesenchymal Stem Cells in Stroke Therapy-From Bench to Bedside. J. Neurological Sci. 352, 1–11. 10.1016/j.jns.2015.03.014 10.1016/j.jns.2015.03.014 | Google Scholar 25818674

[B52] Dyer D. P. Thomson J. M. Hermant A. Jowitt T. A. Handel T. M. Proudfoot A. E. I. (19502014). TSG-6 Inhibits Neutrophil Migration via Direct Interaction with the Chemokine CXCL8. J. I. 192, 2177–2185. 10.4049/jimmunol.1300194 *Md* PubMed Abstract | 10.4049/jimmunol.1300194 | Google Scholar PMC3988464 24501198

[B53] Enstrom A. M. Lit L. Onore C. E. Gregg J. P. Hansen R. L. Pessah I. N. (2009). Altered Gene Expression and Function of Peripheral Blood Natural Killer Cells in Children with Autism. Brain, Behav. Immun. 23, 124–133. 10.1016/j.bbi.2008.08.001 PubMed Abstract | 10.1016/j.bbi.2008.08.001 | Google Scholar 18762240 PMC2636576

[B54] Enstrom A. M. Onore C. E. Van de Water J. A. Ashwood P. (2010). Differential Monocyte Responses to TLR Ligands in Children with Autism Spectrum Disorders. Brain, Behav. Immun. 24, 64–71. 10.1016/j.bbi.2009.08.001 10.1016/j.bbi.2009.08.001 | Google Scholar 19666104 PMC3014091

[B55] Espagnolle N. Balguerie A. Arnaud E. Sensebé L. Varin A. (2017). CD54-Mediated Interaction with Pro-inflammatory Macrophages Increases the Immunosuppressive Function of Human Mesenchymal Stromal Cells. Stem Cell Rep. 8, 961–976. 10.1016/j.stemcr.2017.02.008 PubMed Abstract | 10.1016/j.stemcr.2017.02.008 | Google Scholar PMC5390105 28330617

[B56] Fan L. Hu C. Chen J. Cen P. Wang J. Li L. (2016). Interaction between Mesenchymal Stem Cells and B-Cells. Ijms 17, 650. 10.3390/ijms17050650 PubMed Abstract | 10.3390/ijms17050650 | Google Scholar PMC4881476 27164080

[B57] Fatemi S. H. Aldinger K. A. Ashwood P. Bauman M. L. Blaha C. D. Blatt G. J. (2012). Consensus Paper: Pathological Role of the Cerebellum in Autism. Cerebellum 11, 777–807. 10.1007/s12311-012-0355-9 PubMed Abstract | 10.1007/s12311-012-0355-9 | Google Scholar 22370873 PMC3677555

[B58] Fatemi S. H. Halt A. R. Stary J. M. Realmuto G. M. Jalali-Mousavi M. (2001). Reduction in Anti-apoptotic Protein Bcl-2 in Autistic Cerebellum. Neuroreport 12, 929–933. 10.1097/00001756-200104170-00013 PubMed Abstract | 10.1097/00001756-200104170-00013 | Google Scholar 11303762

[B59] Ferreira L. M. R. Meissner T. B. Tilburgs T. Strominger J. L. (2017). HLA-G: At the Interface of Maternal-Fetal Tolerance. Trends Immunol. 38, 272–286. 10.1016/j.it.2017.01.009 PubMed Abstract | 10.1016/j.it.2017.01.009 | Google Scholar 28279591

[B60] Finegold S. M. Dowd S. E. Gontcharova V. Liu C. Henley K. E. Wolcott R. D. (2010). Pyrosequencing Study of Fecal Microflora of Autistic and Control Children. Anaerobe 16, 444–453. 10.1016/j.anaerobe.2010.06.008 PubMed Abstract | 10.1016/j.anaerobe.2010.06.008 | Google Scholar 20603222

[B61] Fox E. Amaral D. Van de Water J. (2012). Maternal and Fetal Antibrain Antibodies in Development and Disease. Devel Neurobio 72, 1327–1334. 10.1002/dneu.22052 PubMed Abstract | 10.1002/dneu.22052 | Google Scholar PMC3478666 22911883

[B62] François M. Copland I. B. Yuan S. Romieu-Mourez R. Waller E. K. Galipeau J. (2012). Cryopreserved Mesenchymal Stromal Cells Display Impaired Immunosuppressive Properties as a Result of Heat-Shock Response and Impaired Interferon-γ Licensing. Cytotherapy 14, 147–152. 10.3109/14653249.2011.623691 PubMed Abstract | 10.3109/14653249.2011.623691 | Google Scholar 22029655 PMC3279133

[B63] Friedenstein A. J. Gorskaja J. F. Kulagina N. N. (1976). Fibroblast Precursors in Normal and Irradiated Mouse Hematopoietic Organs. Exp. Hematol. 4, 267–274. PubMed Abstract | Google Scholar 976387

[B64] Geschwind D. H. Levitt P. (2007). Autism Spectrum Disorders: Developmental Disconnection Syndromes. Curr. Opin. Neurobiol. 17, 103–111. 10.1016/j.conb.2007.01.009 PubMed Abstract | 10.1016/j.conb.2007.01.009 | Google Scholar 17275283

[B65] Gesundheit B. Ashwood P. Keating A. Naor D. Melamed M. Rosenzweig J. P. (2015). Therapeutic Properties of Mesenchymal Stem Cells for Autism Spectrum Disorders. Med. Hypotheses 84, 169–177. 10.1016/j.mehy.2014.12.016 PubMed Abstract | 10.1016/j.mehy.2014.12.016 | Google Scholar 25592283

[B66] Gesundheit B. Rosenzweig J. P. (2017). Editorial: Autism Spectrum Disorders (ASD)-Searching for the Biological Basis for Behavioral Symptoms and New Therapeutic Targets. Front. Neurosci. 10, 10. 10.3389/fnins.2016.00607 PubMed Abstract | 10.3389/fnins.2016.00607 | Google Scholar PMC5220064 28119558

[B67] Gesundheit B. Rosenzweig J. P. Naor D. Lerer B. Zachor D. A. Procházka V. (2013). Immunological and Autoimmune Considerations of Autism Spectrum Disorders. J. Autoimmun. 44, 1–7. 10.1016/j.jaut.2013.05.005 PubMed Abstract | 10.1016/j.jaut.2013.05.005 | Google Scholar 23867105

[B68] Gieseke F. Schütt B. Viebahn S. Koscielniak E. Friedrich W. Handgretinger R. (2007). Human Multipotent Mesenchymal Stromal Cells Inhibit Proliferation of PBMCs Independently of IFNγR1 Signaling and Ido Expression. Blood 110, 2197–2200. 10.1182/blood-2007-04-083162 PubMed Abstract | 10.1182/blood-2007-04-083162 | Google Scholar 17522338

[B69] Giri J. Das R. Nylen E. Chinnadurai R. Galipeau J. (2020). CCL2 and CXCL12 Derived from Mesenchymal Stromal Cells Cooperatively Polarize IL-10+ Tissue Macrophages to Mitigate Gut Injury. Cell Rep. 30, 1923–1934. e4. 10.1016/j.celrep.2020.01.047 PubMed Abstract | 10.1016/j.celrep.2020.01.047 | Google Scholar 32049021 PMC7043065

[B70] Glennie S. Soeiro I. Dyson P. J. Lam E. W.-F. Dazzi F. (2005). Bone Marrow Mesenchymal Stem Cells Induce Division Arrest Anergy of Activated T Cells. Blood 105, 2821–2827. 10.1182/blood-2004-09-3696 PubMed Abstract | 10.1182/blood-2004-09-3696 | Google Scholar 15591115

[B71] Gomez-Salazar M. Gonzalez-Galofre Z. N. Casamitjana J. Crisan M. James A. W. Péault B. (2020). Five Decades Later, Are Mesenchymal Stem Cells Still Relevant? Front. Bioeng. Biotechnol. 8, 148. 10.3389/fbioe.2020.00148 PubMed Abstract | 10.3389/fbioe.2020.00148 | Google Scholar 32185170 PMC7058632

[B72] Götherström C. Lundqvist A. Duprez I. R. Childs R. Berg L. le Blanc K. (2011). Fetal and Adult Multipotent Mesenchymal Stromal Cells Are Killed by Different Pathways. Cytotherapy 13, 269–278. 10.3109/14653249.2010.523077 PubMed Abstract | 10.3109/14653249.2010.523077 | Google Scholar 20942778

[B73] Grigorenko E. L. Han S. S. Yrigollen C. M. Leng L. Mizue Y. Anderson G. M. (2008). Macrophage Migration Inhibitory Factor and Autism Spectrum Disorders. Pediatrics 122, e438–e445. 10.1542/peds.2007-3604 PubMed Abstract | 10.1542/peds.2007-3604 | Google Scholar 18676531 PMC3816765

[B74] Guerini F. R. Bolognesi E. Chiappedi M. Ghezzo A. Canevini M. P. Mensi M. M. (2015). An HLA-G∗14bp Insertion/deletion Polymorphism Associates with the Development of Autistic Spectrum Disorders. Brain, Behav. Immun. 44, 207–212. 10.1016/j.bbi.2014.10.002 PubMed Abstract | 10.1016/j.bbi.2014.10.002 | Google Scholar 25451607

[B75] Guerini F. R. Bolognesi E. Chiappedi M. Ghezzo A. Manca S. Zanette M. (2018). HLA-G∗14bp Insertion and the KIR2DS1-HLAC2 Complex Impact on Behavioral Impairment in Children with Autism Spectrum Disorders. Neuroscience 370, 163–169. 10.1016/j.neuroscience.2017.06.012 PubMed Abstract | 10.1016/j.neuroscience.2017.06.012 | Google Scholar 28627421

[B76] Guerini F. R. Bolognesi E. Chiappedi M. Ripamonti E. Ghezzo A. Zanette M. (2018). HLA-G Coding Region Polymorphism Is Skewed in Autistic Spectrum Disorders. Brain, Behav. Immun. 67, 308–313. 10.1016/j.bbi.2017.09.007 PubMed Abstract | 10.1016/j.bbi.2017.09.007 | Google Scholar 28923404

[B77] Guerini F. R. Bolognesi E. Sotgiu S. Carta A. Clerici C. Chiappedi M. (2019). HLA-G Allelic Distribution in Sardinian Children with Autism Spectrum Disorders: A Replication Study. Brain, Behav. Immun. 79, 314–318. 10.1016/j.bbi.2019.02.003 PubMed Abstract | 10.1016/j.bbi.2019.02.003 | Google Scholar 30763769

[B78] Guilmatre A. Huguet G. Delorme R. Bourgeron T. (2014). The Emerging Role ofSHANKgenes in Neuropsychiatric Disorders. Devel Neurobio 74, 113–122. 10.1002/dneu.22128 PubMed Abstract | 10.1002/dneu.22128 | Google Scholar 24124131

[B79] Harvey L. Boksa P. (2012). Prenatal and Postnatal Animal Models of Immune Activation: Relevance to a Range of Neurodevelopmental Disorders. Devel Neurobio 72, 1335–1348. 10.1002/dneu.22043 10.1002/dneu.22043 | Google Scholar 22730147

[B80] Heuer L. Braunschweig D. Ashwood P. Van de Water J. Campbell D. B. (2011). Association of a MET Genetic Variant with Autism-Associated Maternal Autoantibodies to Fetal Brain Proteins and Cytokine Expression. Transl. Psychiatry 1, e48. 10.1038/tp.2011.48 PubMed Abstract | 10.1038/tp.2011.48 | Google Scholar 22833194 PMC3309488

[B81] Jaimes Y. Naaldijk Y. Wenk K. Leovsky C. Emmrich F. (2017). Mesenchymal Stem Cell-Derived Microvesicles Modulate Lipopolysaccharides-Induced Inflammatory Responses to Microglia Cells. Stem Cells Dayt Ohio 35, 812–823. 10.1002/stem.2541 PubMed Abstract | 10.1002/stem.2541 | Google Scholar 27862694

[B82] Jiang X.-X. Zhang Y. Liu B. Zhang S.-X. Wu Y. Yu X.-D. (2005). Human Mesenchymal Stem Cells Inhibit Differentiation and Function of Monocyte-Derived Dendritic Cells. Blood 105, 4120–4126. 10.1182/blood-2004-02-0586 PubMed Abstract | 10.1182/blood-2004-02-0586 | Google Scholar 15692068

[B83] Johnson W. G. Buyske S. Mars A. E. Sreenath M. Stenroos E. S. Williams T. A. (2009). HLA-DR4 as a Risk Allele for Autism Acting in Mothers of Probands Possibly during Pregnancy. Arch. Pediatr. Adolesc. Med. 163, 542–546. 10.1001/archpediatrics.2009.74 PubMed Abstract | 10.1001/archpediatrics.2009.74 | Google Scholar 19487610

[B84] Jüttler E. Tarabin V. Schwaninger M. (2002). Interleukin-6 (IL-6): a Possible Neuromodulator Induced by Neuronal Activity. Neuroscientist 8, 268–275. 10.1177/1073858402008003012 PubMed Abstract | 10.1177/1073858402008003012 | Google Scholar 12061506

[B85] Karussis D. Karageorgiou C. Vaknin-Dembinsky A. Gowda-Kurkalli B. Gomori J. M. Kassis I. (2010). Safety and Immunological Effects of Mesenchymal Stem Cell Transplantation in Patients with Multiple Sclerosis and Amyotrophic Lateral Sclerosis. Arch. Neurol. 67, 1187–1194. 10.1001/archneurol.2010.248 PubMed Abstract | 10.1001/archneurol.2010.248 | Google Scholar 20937945 PMC3036569

[B86] Kern S. Eichler H. Stoeve J. Klüter H. Bieback K. (2006). Comparative Analysis of Mesenchymal Stem Cells from Bone Marrow, Umbilical Cord Blood, or Adipose Tissue. Stem Cells 24, 1294–1301. 10.1634/stemcells.2005-0342 PubMed Abstract | 10.1634/stemcells.2005-0342 | Google Scholar 16410387

[B87] King B. H. Navot N. Bernier R. Webb S. J. (2014). Update on Diagnostic Classification in Autism. Curr. Opin. Psychiatry 27, 105–109. 10.1097/YCO.0000000000000040 PubMed Abstract | 10.1097/YCO.0000000000000040 | Google Scholar 24441420 PMC4929984

[B88] Koh S. Kim N. Yin H. H. Harris I. R. Dejneka N. S. Eroglu C. (2015). Human Umbilical Tissue-Derived Cells Promote Synapse Formation and Neurite Outgrowth via Thrombospondin Family Proteins. J. Neurosci. 35, 15649–15665. 10.1523/JNEUROSCI.1364-15.2015 PubMed Abstract | 10.1523/JNEUROSCI.1364-15.2015 | Google Scholar 26609158 PMC4659827

[B89] Krampera M. Cosmi L. Angeli R. Pasini A. Liotta F. Andreini A. (2006). Role for Interferon-γ in the Immunomodulatory Activity of Human Bone Marrow Mesenchymal Stem Cells. Stem Cells Dayt Ohio 24, 386–398. 10.1634/stemcells.2005-0008 10.1634/stemcells.2005-0008 | Google Scholar 16123384

[B90] Lai M-C. Lombardo M. V. Baron-Cohen S. (2014). Autism. Lancet, 383. 10.1016/S0140-6736(13)61539-1 10.1016/S0140-6736(13)61539-1 | Google Scholar 24074734

[B91] Le Blanc K. Davies L. C. (2015). Mesenchymal Stromal Cells and the Innate Immune Response. Immunol. Lett. 168, 140–146. 10.1016/j.imlet.2015.05.004 PubMed Abstract | 10.1016/j.imlet.2015.05.004 | Google Scholar 25982165

[B92] Lee L.-C. Zachary A. A. Leffell M. S. Newschaffer C. J. Matteson K. J. Tyler J. D. (2006). HLA-DR4 in Families with Autism. Pediatr. Neurol. 35, 303–307. 10.1016/j.pediatrneurol.2006.06.006 PubMed Abstract | 10.1016/j.pediatrneurol.2006.06.006 | Google Scholar 17074598

[B93] Lee R. H. Pulin A. A. Seo M. J. Kota D. J. Ylostalo J. Larson B. L. (2009). Intravenous hMSCs Improve Myocardial Infarction in Mice Because Cells Embolized in Lung Are Activated to Secrete the Anti-inflammatory Protein TSG-6. Cell Stem Cell 5, 54–63. 10.1016/j.stem.2009.05.003 PubMed Abstract | 10.1016/j.stem.2009.05.003 | Google Scholar 19570514 PMC4154377

[B94] Li X. Chauhan A. Sheikh A. M. Patil S. Chauhan V. Li X.-M. (2009). Elevated Immune Response in the Brain of Autistic Patients. J. Neuroimmunol. 207, 111–116. 10.1016/j.jneuroim.2008.12.002 PubMed Abstract | 10.1016/j.jneuroim.2008.12.002 | Google Scholar 19157572 PMC2770268

[B95] Libbey J. Sweeten T. McMahon W. Fujinami R. (2005). Autistic Disorder and Viral Infections. J. NeuroVirology 11, 1–10. 10.1080/13550280590900553 10.1080/13550280590900553 | Google Scholar 15804954

[B96] Lintas C. Altieri L. Lombardi F. Sacco R. Persico A. M. (2010). Association of Autism with Polyomavirus Infection in Postmortem Brains. J. Neurovirol 16, 141–149. 10.3109/13550281003685839 PubMed Abstract | 10.3109/13550281003685839 | Google Scholar 20345322

[B97] Liu R. Chang W. Wei H. Zhang K. (2016). Comparison of the Biological Characteristics of Mesenchymal Stem Cells Derived from Bone Marrow and Skin. Stem Cells Int. 2016, 1–12. 10.1155/2016/3658798 PubMed Abstract | 10.1155/2016/3658798 | Google Scholar PMC4863123 27239202

[B98] Lombard J. (1998). Autism: a Mitochondrial Disorder? Med. Hypotheses 50, 497–500. 10.1016/s0306-9877(98)90270-5 PubMed Abstract | 10.1016/s0306-9877(98)90270-5 | Google Scholar 9710323

[B99] Luna R. A. Oezguen N. Balderas M. Venkatachalam A. Runge J. K. Versalovic J. (2017). Distinct Microbiome-Neuroimmune Signatures Correlate with Functional Abdominal Pain in Children with Autism Spectrum Disorder. Cell. Mol. Gastroenterology Hepatology 3, 218–230. 10.1016/j.jcmgh.2016.11.008 PubMed Abstract | 10.1016/j.jcmgh.2016.11.008 | Google Scholar PMC5331780 28275689

[B100] Lv Y.-T. Zhang Y. Liu M. Qiuwaxi J.-n. -t. Ashwood P. Cho S. C. (2013). Transplantation of Human Cord Blood Mononuclear Cells and Umbilical Cord-Derived Mesenchymal Stem Cells in Autism. J. Transl. Med. 11, 196. 10.1186/1479-5876-11-196 PubMed Abstract | 10.1186/1479-5876-11-196 | Google Scholar 23978163 PMC3765833

[B101] Maby-El Hajjami H. Amé-Thomas P. Pangault C. Tribut O. DeVos J. Jean R. (2009). Functional Alteration of the Lymphoma Stromal Cell Niche by the Cytokine Context: Role of Indoleamine-2,3 Dioxygenase. Cancer Res. 69, 3228–3237. 10.1158/0008-5472.CAN-08-3000 PubMed Abstract | 10.1158/0008-5472.CAN-08-3000 | Google Scholar 19276371

[B102] Maggini J. Mirkin G. Bognanni I. Holmberg J. Piazzón I. M. Nepomnaschy I. (2010). Mouse Bone Marrow-Derived Mesenchymal Stromal Cells Turn Activated Macrophages into a Regulatory-like Profile. PloS One 5, e9252. 10.1371/journal.pone.0009252 PubMed Abstract | 10.1371/journal.pone.0009252 | Google Scholar 20169081 PMC2821929

[B103] Malkova N. V. Yu C. Z. Hsiao E. Y. Moore M. J. Patterson P. H. (2012). Maternal Immune Activation Yields Offspring Displaying Mouse Versions of the Three Core Symptoms of Autism. Brain, Behav. Immun. 26, 607–616. 10.1016/j.bbi.2012.01.011 PubMed Abstract | 10.1016/j.bbi.2012.01.011 | Google Scholar 22310922 PMC3322300

[B104] Marquez-Curtis L. A. Janowska-Wieczorek A. McGann L. E. Elliott J. A. W. (2015). Mesenchymal Stromal Cells Derived from Various Tissues: Biological, Clinical and Cryopreservation Aspects. Cryobiology 71, 181–197. 10.1016/j.cryobiol.2015.07.003 PubMed Abstract | 10.1016/j.cryobiol.2015.07.003 | Google Scholar 26186998

[B105] Maximo J. O. Cadena E. J. Kana R. K. (2014). The Implications of Brain Connectivity in the Neuropsychology of Autism. Neuropsychol. Rev. 24, 16–31. 10.1007/s11065-014-9250-0 PubMed Abstract | 10.1007/s11065-014-9250-0 | Google Scholar 24496901 PMC4059500

[B106] Mays R. Deans R. (2016). Adult Adherent Cell Therapy for Ischemic Stroke: Clinical Results and Development Experience Using MultiStem. Transfusion 56, 6S–8S. 10.1111/trf.13562 PubMed Abstract | 10.1111/trf.13562 | Google Scholar 27079323

[B107] Meltzer A. Van de Water J. (2017). The Role of the Immune System in Autism Spectrum Disorder. Neuropsychopharmacol 42, 284–298. 10.1038/npp.2016.158 PubMed Abstract | 10.1038/npp.2016.158 | Google Scholar PMC5143489 27534269

[B108] Menard C. Pacelli L. Bassi G. Dulong J. Bifari F. Bezier I. (2013). Clinical-grade Mesenchymal Stromal Cells Produced under Various Good Manufacturing Practice Processes Differ in Their Immunomodulatory Properties: Standardization of Immune Quality Controls. Stem Cells Dev. 22, 1789–1801. 10.1089/scd.2012.0594 PubMed Abstract | 10.1089/scd.2012.0594 | Google Scholar 23339531 PMC3668498

[B109] Mendes Filho D. Ribeiro P. d. C. Oliveira L. F. de Paula D. R. M. Capuano V. de Assunção T. S. F. (2018). Therapy with Mesenchymal Stem Cells in Parkinson Disease: History and Perspectives. Neurologist 23, 141–147. 10.1097/NRL.0000000000000188 PubMed Abstract | 10.1097/NRL.0000000000000188 | Google Scholar 29953040

[B110] Mohyeddin Bonab M. Ali Sahraian M. Aghsaie A. Ahmadi Karvigh S. Massoud Hosseinian S. Nikbin B. (2012). Autologous Mesenchymal Stem Cell Therapy in Progressive Multiple Sclerosis: an Open Label Study. Cscr 7, 407–414. 10.2174/157488812804484648 10.2174/157488812804484648 | Google Scholar 23061813

[B111] Molloy C. Morrow A. Meinzenderr J. Schleifer K. Dienger K. Manningcourtney P. (2006). Elevated Cytokine Levels in Children with Autism Spectrum Disorder. J. Neuroimmunol. 172, 198–205. 10.1016/j.jneuroim.2005.11.007 PubMed Abstract | 10.1016/j.jneuroim.2005.11.007 | Google Scholar 16360218

[B112] Money J. Bobrow N. A. Clarke F. C. (1971). Autism and Autoimmune Disease: a Family Study. J. Autism Dev. Disord. 1, 146–160. 10.1007/BF01537954 PubMed Abstract | 10.1007/BF01537954 | Google Scholar 5172389

[B113] Murias M. Major S. Compton S. Buttinger J. Sun J. M. Kurtzberg J. (2018). Electrophysiological Biomarkers Predict Clinical Improvement in an Open-Label Trial Assessing Efficacy of Autologous Umbilical Cord Blood for Treatment of Autism. Stem Cells Transl. Med. 7, 783–791. 10.1002/sctm.18-0090 PubMed Abstract | 10.1002/sctm.18-0090 | Google Scholar 30070044 PMC6216432

[B114] Nauta A. J. Kruisselbrink A. B. Lurvink E. Willemze R. Fibbe W. E. (2006). Mesenchymal Stem Cells Inhibit Generation and Function of Both CD34+-Derived and Monocyte-Derived Dendritic Cells. J. Immunol. Md 177, 2080–2087. 10.4049/jimmunol.177.4.2080 10.4049/jimmunol.177.4.2080 | Google Scholar 16887966

[B115] Németh K. Leelahavanichkul A. Yuen P. S. T. Mayer B. Parmelee A. Doi K. (2009). Bone Marrow Stromal Cells Attenuate Sepsis via Prostaglandin E(2)-dependent Reprogramming of Host Macrophages to Increase Their Interleukin-10 Production. Nat. Med. 15, 42–49. 10.1038/nm.1905 PubMed Abstract | 10.1038/nm.1905 | Google Scholar 19098906 PMC2706487

[B116] Nicolson G. L. Gan R. Nicolson N. L. Haier J. (2007). Evidence for Mycoplasma ssp.,Chlamydia Pneunomiae, and Human Herpes Virus-6 Coinfections in the Blood of Patients with Autistic Spectrum Disorders. J. Neurosci. Res. 85, 1143–1148. 10.1002/jnr.21203 PubMed Abstract | 10.1002/jnr.21203 | Google Scholar 17265454

[B117] Noriega D. B. Savelkoul H. F. J. (2014). Immune Dysregulation in Autism Spectrum Disorder. Eur. J. Pediatr. 173, 33–43. 10.1007/s00431-013-2183-4 PubMed Abstract | 10.1007/s00431-013-2183-4 | Google Scholar 24297668

[B118] Odell D. Maciulis A. Cutler A. Warren L. McMahon W. M. Coon H. (2005). Confirmation of the Association of the C4B Null Allelle in Autism. Hum. Immunol. 66, 140–145. 10.1016/j.humimm.2004.11.002 PubMed Abstract | 10.1016/j.humimm.2004.11.002 | Google Scholar 15694999

[B119] Onore C. Careaga M. Ashwood P. (2012). The Role of Immune Dysfunction in the Pathophysiology of Autism. Brain, Behav. Immun. 26, 383–392. 10.1016/j.bbi.2011.08.007 10.1016/j.bbi.2011.08.007 | Google Scholar 21906670 PMC3418145

[B120] Ooi Y. Y. Dheen S. T. Sam Wah Tay S. (2015). Paracrine Effects of Mesenchymal Stem Cells-Conditioned Medium on Microglial Cytokines Expression and Nitric Oxide Production. Neuroimmunomodulation 22, 233–242. 10.1159/000365483 PubMed Abstract | 10.1159/000365483 | Google Scholar 25341618

[B121] Patel S. A. Meyer J. R. Greco S. J. Corcoran K. E. Bryan M. Rameshwar P. (2010). Mesenchymal Stem Cells Protect Breast Cancer Cells through Regulatory T Cells: Role of Mesenchymal Stem Cell-Derived TGF-β. J.I. Md 184, 5885–5894. 10.4049/jimmunol.0903143 10.4049/jimmunol.0903143 | Google Scholar 20382885

[B122] Perets N. Hertz S. London M. Offen D. (2018). Intranasal Administration of Exosomes Derived from Mesenchymal Stem Cells Ameliorates Autistic-like Behaviors of BTBR Mice. Mol. Autism 9, 57. 10.1186/s13229-018-0240-6 PubMed Abstract | 10.1186/s13229-018-0240-6 | Google Scholar 30479733 PMC6249852

[B123] Perets N. Oron O. Herman S. Elliott E. Offen D. (2020). Exosomes Derived from Mesenchymal Stem Cells Improved Core Symptoms of Genetically Modified Mouse Model of Autism Shank3B. Mol. Autism 11, 65. 10.1186/s13229-020-00366-x PubMed Abstract | 10.1186/s13229-020-00366-x | Google Scholar 32807217 PMC7433169

[B124] Phillips M. Pozzo-Miller L. (2015). Dendritic Spine Dysgenesis in Autism Related Disorders. Neurosci. Lett. 601, 30–40. 10.1016/j.neulet.2015.01.011 PubMed Abstract | 10.1016/j.neulet.2015.01.011 | Google Scholar 25578949 PMC4496332

[B125] Plioplys A. V. Greaves A. Kazemi K. Silverman E. (1994). Lymphocyte Function in Autism and Rett Syndrome. Neuropsychobiology 29, 12–16. 10.1159/000119056 PubMed Abstract | 10.1159/000119056 | Google Scholar 8127418

[B126] Polchert D. Sobinsky J. Douglas G. Kidd M. Moadsiri A. Reina E. (2008). IFN-γ Activation of Mesenchymal Stem Cells for Treatment and Prevention of Graft versus Host Disease. Eur. J. Immunol. 38, 1745–1755. 10.1002/eji.200738129 PubMed Abstract | 10.1002/eji.200738129 | Google Scholar 18493986 PMC3021120

[B127] Pons R. Andreu A. L. Checcarelli N. Vilà M. R. Engelstad K. Sue C. M. (2004). Mitochondrial DNA Abnormalities and Autistic Spectrum Disorders. J. Pediatr. 144, 81–85. 10.1016/j.jpeds.2003.10.023 PubMed Abstract | 10.1016/j.jpeds.2003.10.023 | Google Scholar 14722523

[B128] Prevosto C. Zancolli M. Canevali P. Zocchi M. R. Poggi A. (2007). Generation of CD4+ or CD8+ Regulatory T Cells upon Mesenchymal Stem Cell-Lymphocyte Interaction. Haematologica 92, 881–888. 10.3324/haematol.11240 PubMed Abstract | 10.3324/haematol.11240 | Google Scholar 17606437

[B129] Price J. (2020). Cell Therapy Approaches to Autism: a Review of Clinical Trial Data. Mol. Autism 11, 37. 10.1186/s13229-020-00348-z PubMed Abstract | 10.1186/s13229-020-00348-z | Google Scholar 32448347 PMC7245880

[B130] Ramasamy R. Fazekasova H. Lam E. W.-F. Soeiro I. s. Lombardi G. Dazzi F. (2007). Mesenchymal Stem Cells Inhibit Dendritic Cell Differentiation and Function by Preventing Entry into the Cell Cycle. Transplantation 83, 71–76. 10.1097/01.tp.0000244572.24780.54 PubMed Abstract | 10.1097/01.tp.0000244572.24780.54 | Google Scholar 17220794

[B131] Remes Lenicov F. Paletta A. L. Gonzalez Prinz M. Varese A. Pavillet C. E. Lopez MaliziaÁ Á. (2018). Prostaglandin E2 Antagonizes TGF-β Actions during the Differentiation of Monocytes into Dendritic Cells. Front. Immunol. 9, 1441. 10.3389/fimmu.2018.01441 PubMed Abstract | 10.3389/fimmu.2018.01441 | Google Scholar 29988364 PMC6023975

[B132] Ren G. Zhang L. Zhao X. Xu G. Zhang Y. Roberts A. I. (2008). Mesenchymal Stem Cell-Mediated Immunosuppression Occurs via Concerted Action of Chemokines and Nitric Oxide. Cell Stem Cell 2, 141–150. 10.1016/j.stem.2007.11.014 PubMed Abstract | 10.1016/j.stem.2007.11.014 | Google Scholar 18371435

[B133] Ren G. Zhao X. Zhang L. Zhang J. L'Huillier A. Ling W. (19502010). Inflammatory Cytokine-Induced Intercellular Adhesion Molecule-1 and Vascular Cell Adhesion Molecule-1 in Mesenchymal Stem Cells Are Critical for Immunosuppression. J. I. Md 184, 2321–2328. 10.4049/jimmunol.0902023 10.4049/jimmunol.0902023 | Google Scholar PMC2881946 20130212

[B134] Ribeiro A. Laranjeira P. Mendes S. Velada I. Leite C. Andrade P. (2013). Mesenchymal Stem Cells from Umbilical Cord Matrix, Adipose Tissue and Bone Marrow Exhibit Different Capability to Suppress Peripheral Blood B, Natural Killer and T Cells. Stem Cell Res. Ther. 4, 125. 10.1186/scrt336 PubMed Abstract | 10.1186/scrt336 | Google Scholar 24406104 PMC3854702

[B135] Rosado M. M. Bernardo M. E. Scarsella M. Conforti A. Giorda E. Biagini S. (2015). Inhibition of B-Cell Proliferation and Antibody Production by Mesenchymal Stromal Cells Is Mediated by T Cells. Stem Cells Dev. 24, 93–103. 10.1089/scd.2014.0155 PubMed Abstract | 10.1089/scd.2014.0155 | Google Scholar 25036865 PMC4273193

[B136] Rylaarsdam L. Guemez-Gamboa A. (2019). Genetic Causes and Modifiers of Autism Spectrum Disorder. Front. Cell. Neurosci. 13, 385. 10.3389/fncel.2019.00385 PubMed Abstract | 10.3389/fncel.2019.00385 | Google Scholar 31481879 PMC6710438

[B137] Sandler R. H. Finegold S. M. Bolte E. R. Buchanan C. P. Maxwell A. P. Väisänen M.-L. (2000). Short-term Benefit from Oral Vancomycin Treatment of Regressive-Onset Autism. J. Child. Neurol. 15, 429–435. 10.1177/088307380001500701 PubMed Abstract | 10.1177/088307380001500701 | Google Scholar 10921511

[B138] Scott-Van Zeeland A. A. Abrahams B. S. Alvarez-Retuerto A. I. Sonnenblick L. I. Rudie J. D. Ghahremani D. (2010). Altered Functional Connectivity in Frontal Lobe Circuits Is Associated with Variation in the Autism Risk Gene CNTNAP2. Sci. Transl. Med. 2, 56ra80. 10.1126/scitranslmed.3001344 PubMed Abstract | 10.1126/scitranslmed.3001344 | Google Scholar PMC3065863 21048216

[B139] Segal-Gavish H. Karvat G. Barak N. Barzilay R. Ganz J. Edry L. (2016). Mesenchymal Stem Cell Transplantation Promotes Neurogenesis and Ameliorates Autism Related Behaviors in BTBR Mice. Autism Res. 9, 17–32. 10.1002/aur.1530 PubMed Abstract | 10.1002/aur.1530 | Google Scholar 26257137

[B140] Selmani Z. Naji A. Zidi I. Favier B. Gaiffe E. Obert L. (2008). Human Leukocyte Antigen-G5 Secretion by Human Mesenchymal Stem Cells Is Required to Suppress T Lymphocyte and Natural Killer Function and to Induce CD4+CD25highFOXP3+ Regulatory T Cells. Stem Cells Dayt Ohio 26, 212–222. 10.1634/stemcells.2007-0554 10.1634/stemcells.2007-0554 | Google Scholar 17932417

[B141] Shahaduzzaman M. Golden J. E. Green S. Gronda A. E. Adrien E. Ahmed A. (2013). A Single Administration of Human Umbilical Cord Blood T Cells Produces Long-Lasting Effects in the Aging hippocampus. Age 35, 2071–2087. 10.1007/s11357-012-9496-5 PubMed Abstract | 10.1007/s11357-012-9496-5 | Google Scholar 23263793 PMC3825009

[B142] Sheikh A. M. Malik M. Wen G. Chauhan A. Chauhan V. Gong C.-X. (2010). BDNF-Akt-Bcl2 Antiapoptotic Signaling Pathway Is Compromised in the Brain of Autistic Subjects. J. Neurosci. Res. 88, a–n. 10.1002/jnr.22416 PubMed Abstract | 10.1002/jnr.22416 | Google Scholar 20648653

[B143] Simhal A. K. Carpenter K. L. H. Nadeem S. Kurtzberg J. Song A. Tannenbaum A. (2020). Measuring Robustness of Brain Networks in Autism Spectrum Disorder with Ricci Curvature. Sci. Rep. 10, 10. 10.1038/s41598-020-67474-9 PubMed Abstract | 10.1038/s41598-020-67474-9 | Google Scholar 32616759 PMC7331646

[B144] Siniscalco D. Bradstreet J. J. Sych N. Antonucci N. (2014). Mesenchymal Stem Cells in Treating Autism: Novel Insights. Wjsc 6, 173–178. 10.4252/wjsc.v6.i2.173 PubMed Abstract | 10.4252/wjsc.v6.i2.173 | Google Scholar 24772244 PMC3999775

[B145] Sioud M. Mobergslien A. Boudabous A. Fløisand Y. (2011). Mesenchymal Stem Cell-Mediated T Cell Suppression Occurs through Secreted Galectins. Int. J. Oncol. 38, 385–390. 10.3892/ijo.2010.869 PubMed Abstract | 10.3892/ijo.2010.869 | Google Scholar 21165555

[B146] Sotiropoulou P. A. Perez S. A. Gritzapis A. D. Baxevanis C. N. Papamichail M. (2006). Interactions between Human Mesenchymal Stem Cells and Natural Killer Cells. Stem Cells Dayt Ohio 24, 74–85. 10.1634/stemcells.2004-0359 10.1634/stemcells.2004-0359 | Google Scholar 16099998

[B147] Spaggiari G. M. Capobianco A. Abdelrazik H. Becchetti F. Mingari M. C. Moretta L. (2008). Mesenchymal Stem Cells Inhibit Natural Killer-Cell Proliferation, Cytotoxicity, and Cytokine Production: Role of Indoleamine 2,3-dioxygenase and Prostaglandin E2. Blood 111, 1327–1333. 10.1182/blood-2007-02-074997 PubMed Abstract | 10.1182/blood-2007-02-074997 | Google Scholar 17951526

[B148] Spees J. L. Lee R. H. Gregory C. A. (2016). Mechanisms of Mesenchymal Stem/stromal Cell Function. Stem Cell Res. Ther. 7, 125. 10.1186/s13287-016-0363-7 PubMed Abstract | 10.1186/s13287-016-0363-7 | Google Scholar 27581859 PMC5007684

[B149] Stubbs E. G. (1976). Autistic Children Exhibit Undetectable Hemagglutination-Inhibition Antibody Titers Despite Previous Rubella Vaccination. J. Autism Dev. Disord. 6, 269–274. 10.1007/BF01543467 10.1007/BF01543467 | Google Scholar 1036494

[B150] Stubbs E. G. (1978). Autistic Symptoms in a Child with Congenital Cytomegaloviras Infection. J. Autism Dev. Disord. 8, 37–43. 10.1007/BF01550276 PubMed Abstract | 10.1007/BF01550276 | Google Scholar 205531

[B151] Sun J. M. Dawson G. Franz L. Howard J. McLaughlin C. Kistler B. (2020). Infusion of Human Umbilical Cord Tissue Mesenchymal Stromal Cells in Children with Autism Spectrum Disorder. Stem Cells Transl. Med. 9, 1137–1146. 10.1002/sctm.19-0434 PubMed Abstract | 10.1002/sctm.19-0434 | Google Scholar 32531111 PMC7519773

[B152] Terraza-Aguirre C. Campos-Mora M. Elizondo-Vega R. Contreras-López R. A. Luz-Crawford P. Jorgensen C. (2020). Mechanisms behind the Immunoregulatory Dialogue between Mesenchymal Stem Cells and Th17 Cells. Cells 9, 1660. 10.3390/cells9071660 PubMed Abstract | 10.3390/cells9071660 | Google Scholar PMC7408034 32664207

[B153] Thompson Foundation for Autism (2012). Autism Spectrum Disorders: Guide to Evidence-Based Interventions. Available at: https://autismguidelines.dmh.mo.gov/documents/Interventions.pdf . Google Scholar

[B154] Thompson M. Mei S. H. J. Wolfe D. Champagne J. Fergusson D. Stewart D. J. (2020). Cell Therapy with Intravascular Administration of Mesenchymal Stromal Cells Continues to Appear Safe: An Updated Systematic Review and Meta-Analysis. EClinicalMedicine 19, 100249. 10.1016/j.eclinm.2019.100249 PubMed Abstract | 10.1016/j.eclinm.2019.100249 | Google Scholar 31989101 PMC6970160

[B155] Tse W. T. Pendleton J. D. Beyer W. M. Egalka M. C. Guinan E. C. (2003). Suppression of Allogeneic T-Cell Proliferation by Human Marrow Stromal Cells: Implications in Transplantation. Transplantation 75, 389–397. 10.1097/01.tp.0000045055.63901.a9 PubMed Abstract | 10.1097/01.tp.0000045055.63901.a9 | Google Scholar 12589164

[B156] Vaags A. K. Lionel A. C. Sato D. Goodenberger M. Stein Q. P. Curran S. (2012). Rare Deletions at the Neurexin 3 Locus in Autism Spectrum Disorder. Am. J. Hum. Genet. 90, 133–141. 10.1016/j.ajhg.2011.11.025 PubMed Abstract | 10.1016/j.ajhg.2011.11.025 | Google Scholar 22209245 PMC3257896

[B157] Vargas D. L. Nascimbene C. Krishnan C. Zimmerman A. W. Pardo C. A. (2005). Neuroglial Activation and Neuroinflammation in the Brain of Patients with Autism. Ann. Neurol. 57, 67–81. 10.1002/ana.20315 PubMed Abstract | 10.1002/ana.20315 | Google Scholar 15546155

[B158] Voineagu I. Wang X. Johnston P. Lowe J. K. Tian Y. Horvath S. (2011). Transcriptomic Analysis of Autistic Brain Reveals Convergent Molecular Pathology. Nature 474, 380–384. 10.1038/nature10110 PubMed Abstract | 10.1038/nature10110 | Google Scholar 21614001 PMC3607626

[B159] Vojdani A. Campbell A. W. Anyanwu E. Kashanian A. Bock K. Vojdani E. (2002). Antibodies to Neuron-specific Antigens in Children with Autism: Possible Cross-Reaction with Encephalitogenic Proteins from Milk, Chlamydia Pneumoniae and Streptococcus Group A. J. Neuroimmunol. 129, 168–177. 10.1016/s0165-5728(02)00180-7 PubMed Abstract | 10.1016/s0165-5728(02)00180-7 | Google Scholar 12161033

[B160] Warren R. Singh V. Cole P. Odell J. D. Pingree C. Warren W. L. (1992). Possible Association of the Exetended MHC Haplotype B44-SC30-DR4 with Autism. Immunogenetics 36, 203–207. 10.1007/BF00215048 PubMed Abstract | 10.1007/BF00215048 | Google Scholar 1639438

[B161] Wei H. Alberts I. Li X. (2013). Brain IL-6 and Autism. Neuroscience 252, 320–325. 10.1016/j.neuroscience.2013.08.025 PubMed Abstract | 10.1016/j.neuroscience.2013.08.025 | Google Scholar 23994594

[B162] Williams B. L. Hornig M. Buie T. Bauman M. L. Cho Paik M. Wick I. (2011). Impaired Carbohydrate Digestion and Transport and Mucosal Dysbiosis in the Intestines of Children with Autism and Gastrointestinal Disturbances. PloS One 6, e24585. 10.1371/journal.pone.0024585 PubMed Abstract | 10.1371/journal.pone.0024585 | Google Scholar 21949732 PMC3174969

[B163] World Health Organization (2021). Autism Spectrum Disorders. Geneva, Switzerland: World Health Organization. Available at: https://www.who.int/news-room/fact-sheets/detail/autism-spectrum-disorders (Accessed April 23, 2021). Google Scholar

[B164] Xu M. Xu X. Li J. Li F. (2019). Association between Gut Microbiota and Autism Spectrum Disorder: A Systematic Review and Meta-Analysis. Front. Psychiatry 10, 473. 10.3389/fpsyt.2019.00473 PubMed Abstract | 10.3389/fpsyt.2019.00473 | Google Scholar 31404299 PMC6673757

[B165] Yamout B. Hourani R. Salti H. Barada W. El-Hajj T. Al-Kutoubi A. (2010). Bone Marrow Mesenchymal Stem Cell Transplantation in Patients with Multiple Sclerosis: a Pilot Study. J. Neuroimmunol. 227, 185–189. 10.1016/j.jneuroim.2010.07.013 PubMed Abstract | 10.1016/j.jneuroim.2010.07.013 | Google Scholar 20728948

[B166] Yang S.-H. Park M.-J. Yoon I.-H. Kim S.-Y. Hong S.-H. Shin J.-Y. (2009). Soluble Mediators from Mesenchymal Stem Cells Suppress T Cell Proliferation by Inducing IL-10. Exp. Mol. Med. 41, 315–324. 10.3858/emm.2009.41.5.035 PubMed Abstract | 10.3858/emm.2009.41.5.035 | Google Scholar 19307751 PMC2701980

[B167] Zhang B. Yeo R. W. Y. Lai R. C. Sim E. W. K. Chin K. C. Lim S. K. (2018). Mesenchymal Stromal Cell Exosome-Enhanced Regulatory T-Cell Production through an Antigen-Presenting Cell-Mediated Pathway. Cytotherapy 20, 687–696. 10.1016/j.jcyt.2018.02.372 PubMed Abstract | 10.1016/j.jcyt.2018.02.372 | Google Scholar 29622483

